# Investigating rate-limited sorption, sorption to air–water interfaces, and colloid-facilitated transport during PFAS leaching

**DOI:** 10.1007/s11356-023-30811-2

**Published:** 2023-11-13

**Authors:** Thomas Bierbaum, Scott K. Hansen, Bikash Poudel, Claus Haslauer

**Affiliations:** 1 Research Facility for Subsurface Remediation (VEGAS), University of Stuttgart, Institute for Modelling Environmental Systems (IWS), Pfaffenwaldring 61, 70569 Stuttgart, Germany; 2 Ben-Gurion University of the Negev, Zuckerberg Institute for Water Research, 8499000 Midreshet Ben-Gurion, Israel

**Keywords:** PFAS, Random walk particle tracking, Rate-limited sorption, Air–water interfaces, Colloid-facilitated transport

## Abstract

Various sorption processes affect leaching of per- and polyfluoroalkyl substances (PFAS) such as PFOA and PFOS. The objectives of this study are to (1) compare rate-limited leaching in column and lysimeter experiments, (2) investigate the relevance of sorption to air–water interfaces (AWI), and (3) examine colloid-facilitated transport as a process explaining early experimental breakthrough. A continuum model (CM) with two-domain sorption is used to simulate equilibrium and rate-limited sorption. A random walk particle tracking (PT) model was developed and applied to analyze complex leaching characteristics. Results show that sorption parameters derived from column experiments underestimate long-term PFOA leaching in lysimeter experiments due to early depletion, suggesting that transformation of precursors contributes to the observed long-term leaching in the lysimeters (approximately 0.003 µg/kg/d PFOA). Both models demonstrate that sorption to AWI is the dominant retention mechanism for PFOS in lysimeter experiments, with retardation due to AWI being 3 (CM) to 3.7 (PT) times higher than retardation due to solid phase sorption. Notably, despite a simplified conception of AWI sorption, the PT results are closer to the observations. The PT simulations demonstrate possible colloid-facilitated transport at early time; however, results using substance-specific varying transport parameters align better with the observations, which should be equal if colloid-facilitated transport without additional kinetics is the sole mechanism affecting early breakthrough. Possibly, rate-limited sorption to AWI is relevant during the early stages of the lysimeter experiment. Our findings demonstrate that rate-limited sorption is less relevant for long-term leaching under field conditions compared to transformation of precursors and that sorption to AWI can be the dominant retention mechanism on contaminated sites. Moreover, they highlight the potential of random walk particle tracking as a practical alternative to continuum models for estimating the relative contributions of various retention mechanisms.

**Supplementary Information:**

The online version contains supplementary material available at 10.1007/s11356-023-30811-2.

## Introduction

Per- and polyfluoroalkyl substances (PFAS) are synthetic chemicals with a large variety of applications. PFAS became a global environmental problem (Buck et al. 2011) due to their toxicity (Steenland et al. 2010), persistency (Kotthoff et al. 2020), and bio-accumulative potential (Stahl et al. 2011). The class of PFAS comprises thousands of individual compounds (Organisation for Economic Co-operation and Development 2018). PFOA and PFOS among the perfluoroalkyl acids (PFAAs) are the most important individual PFAS, primarily due to their prevalence at contaminated sites (including the one of this study) and their persistent nature (Martin et al. 2010; Lyu et al. 2018; Bräunig et al. 2019). The transport of PFAS in soil and groundwater is complex due to varying mobility properties of the individual PFAS and various relevant sorption mechanisms (Higgins and Luthy 2006; Campos Pereira et al. 2018). Hydrophobic (Du et al. 2014; Cai et al. 2022) and electrostatic (Campos Pereira et al. 2018; Nguyen et al. 2020) interactions are widely considered the dominant sorption mechanisms to solid phases. Thereby, sorption due to hydrophobic interaction increases with the chain length of PFAAs and with the soils’ content of organic matter (Guelfo and Higgins 2013; Milinovic et al. 2015). Electrostatic interactions are more dominant for short-chain PFAAs as well as cationic and zwitterionic PFAS (Hellsing et al. 2016; Xiao et al. 2019).

Several experimental (Guelfo et al. 2020; Maizel et al. 2021) and modeling studies (Brusseau et al. 2019a; Guelfo et al. 2020) have reported non-ideal transport behavior of PFAS which results in asymmetric breakthrough curves and pronounced tailing. This behavior is often attributed to rate-limited or non-equilibrium sorption, indicating that sorption acts as a kinetic process rather than an instantaneous equilibrium (Brusseau et al. 2019a). To numerically replicate non-ideal transport, many studies employ dual domain sorption models, in which PFAS partitioning between solid and liquid phases is partly modeled rate limited, following a first-order kinetic rate (Lv et al. 2018; Lyu et al. 2018; Brusseau et al. 2019a). Although several studies propose distinct mechanisms for equilibrium sorption and rate-limited sorption, the underlying physicochemical processes remain not fully understood. Electrostatic interactions are generally faster than hydrophobic interactions (Nguyen et al. 2020; Guelfo et al. 2020), with the latter potentially controlling rate-limited leaching (Nguyen et al. 2022). An alternative explanation for rate-limited leaching could be intraparticle or intra-organic matter diffusion (Brusseau et al. 1991). If PFAA precursors are present, transformation to PFAAs (Lee et al. 2014; Liu and Liu 2016, Weidemann et al. 2022) may lead to similar leaching characteristics, i.e., extensive tailing and long-term leaching at fairly low concentrations.

Due to the surface activity of PFAS, sorption to air–water interfaces (AWI) influences their transport in variably saturated conditions. Brusseau (2018) demonstrated that sorption to AWI potentially dominates the retardation of PFOS at concentrations < 1 mg/L. The surface activity of PFAAs, and consequently the sorption affinity to AWI, increases with increasing chain length (Vecitis et al. 2008; Brusseau and van Glubt 2019; Schaefer et al. 2019; Abraham et al. 2022). Various methods exist to describe the relation between saturation and the AWI area (Silva et al. 2022b), all showing that the AWI area increases with decreasing saturation. In other studies, both Freundlich (Schaefer et al. 2019) and Langmuir-type (Silva et al. 2020) isotherms were used to simulate PFAS sorption to AWI. Partitioning to AWI is generally concentration dependent and becoming non-linear with increasing concentrations (Brusseau 2018; Silva et al. 2020). The majority of studies considered sorption to AWI to be instantaneous (Brusseau 2020; Silva et al. 2020). Contrarily, Stults et al. (2023) propose that sorption to AWI may be influenced by rate limitations.

On contaminated sites, extreme precipitation events or disturbance of the soil structure may initiate colloid-facilitated transport and enhanced PFAS leaching. Shao et al. (2016) proposed that observed elevated concentrations of long-chain PFAS were caused by suspended particles and adsorbed PFAS. Contrary to other studies (Guelfo et al. 2020; Maizel et al. 2021), Borthakur et al. (2021) observed elevated leachate concentrations after flow interruption which was explained by mobilization of colloids. This was confirmed by particle size analyses of the leachate samples. They state that the relevance of colloid-facilitated transport is higher for long-chain PFAAs due to their higher sorption affinity.

While Eulerian models are frequently employed for simulating PFAS transport, Lagrangian random walk particle tracking models have not yet been utilized. However, they could potentially serve as a flexible and effective alternative (Dentz and Berkowitz 2003, Loschko et al. 2016; Henri and Diamantopoulos 2022). The movement of individual particles or solutes is determined by advective and dispersive transport terms, and stochastic processes are used to model mass transfer between mobile and immobile phases (Haggerty and Gorelick 1995; Hedia et al. 2021). Generally, this method may be advantageous for simulating solute transport in complex flow fields or in heterogeneous porous media, where traditional continuum models may be limited by numerical dispersion (Boso et al. 2013). In the context of PFAS modeling specifically, particle tracking models may provide enhanced flexibility for examining intricate leaching characteristics involving diverse retention processes.

This study uses a random walk particle tracking model as a practical tool to investigate complex leaching behavior. We developed and applied a random walk particle tracking algorithm including sorption to AWI, sorption to the solid matrix, and colloid-facilitated transport. To the best of our knowledge, this is the first study to simulate PFAS leaching and retention using particle tracking, with the transition of PFAS between mobile and immobile phases. Results of state-of-the-art continuum models and random walk particle tracking are compared. The contribution of this study lies in the incorporation of various retention mechanisms and the demonstration of an alternative simulation approach, providing a comprehensive understanding of the complex PFAS leaching behavior. Moreover, we incorporate the most relevant retention mechanisms, including equilibrium and rate-limited sorption to solid phases, as well as partitioning to AWI, and discuss the contribution of precursors to long-term leaching of PFAAs. Additionally, we explore colloid-facilitated transport as a potential cause for observed premature PFAS leaching. Thus, we present a comprehensive investigation into the leaching characteristics of PFOA and PFOS in both saturated and variably saturated experiments, building on previous work reported by Bierbaum et al. (2023).

## Materials and methods

### Experimental data

The experimental data used in this study was presented in Bierbaum et al. (2023). They investigated PFAS leaching from agricultural topsoil polluted with paper-fiber biosolids. Various PFAAs as well as precursors were detected in the soil: The total concentration of 36 individual PFAS was 2 mg/kg. The soil concentrations of PFOA and PFOS measured by methanolic extraction were 45 µg/kg and 186 µg/kg, respectively. They also investigated leaching from soils treated with immobilization products; however, in the present study, only leaching data of their untreated original soil (N-1) was used (Table 1).

**Table 1:** Overview of the used parameters and the corresponding units

Symbol	Parameter	Unit
*α*	First-order kinetic rate of rate-limited sorption	1/T
*α*_L_	Longitudinal dispersivity	L
*β*	Freundlich exponent	–
*γ*	Air–water surface tension	M/T^2^
*Γ*	Solute concentration on air–water interface	M/L^2^
*λ*_ia_	Adsorption rate to air–water interface	1/T
*λ*_s_	Adsorption rate to solid phase	1/T
*µ*_ia_	Desorption rate from air–water interface	1/T
*µ*_s_	Desorption rate from solid phase	1/T
*ρ*_b_	Bulk density	M/L^3^
*ρ*_w_	Density of water	M/L^3^
*θ*	Water content	L^3^/L^3^
*θ*_s_	Water content at full saturation	L^3^/L^3^
*A*_ia_	Air–water interfacial area	L^2^/L^3^
*c*	Solute concentration in liquid phase	M/L^3^
*c*_0_	Initial solute concentration in liquid phase	M/L^3^
*g*	Gravitational acceleration	L/T^2^
*h*	Hydraulic head	L
*K*_d_	Partitioning coefficient between liquid and solid phases	L^3^/M
*K*_ia_	Partitioning coefficient between liquid phase and air–water interface	L or L^3^/L^2^
LS	Liquid-to-solid ratio	L^3^/M
*m*_s_	Mass of soil	M
*m*_tot_	Total mass of solute	M
*n*	Porosity	L^3^/L^3^
*q*	Discharge	L/T
*R*	Retardation coefficient	–
*s*	Solute concentration on solid phase	M/M
*s*_e_	Sorbed concentration in equilibrium phase	M/M
*s*_k_	Sorbed concentration in kinetic phase	M/M
*s*_w_	Water saturation	–
*t*	Time	T
*v*	Flow velocity	L/T
*V*_p_	Pore Volume	L^3^/L^3^
*x*_n_	Position of particle at time step n (random walk particle tracking)	–

The leaching data used in this study was obtained from four saturated column experiments and one variably saturated lysimeter experiment with relatively long experimental operating times (≈ 100 d for column experiments, ≈ 30 months for lysimeters) presented by Bierbaum et al. (2023). Cumulative leachate samples were taken, i.e., the leachate was cumulated in a sampling container within a sampling interval. In the column experiments, the sampling intervals were initially 2 to 3 d and were increased to about 2 weeks. In the lysimeter experiment, the sampling intervals were initially 2 weeks and were increased to 4 weeks. This concept was followed due to decreasing concentration changes with experimental time. At the end of a sampling interval, a sample of the cumulated leachate was taken and analyzed. Leachate concentrations were measured by target analysis using liquid chromatography–tandem mass spectrometry.

The total masses, *m*_tot_, observed in the individual experiments (sum of the leached mass, *m*_out_, and the residual mass in soil) varied between 39.6 µg/kg PFOA and 48.6 µg/kg PFOA and 108.2 µg/kg PFOS and 205 µg/kg PFOS (Table S1). Residual soil concentrations of PFOA and PFOS were low (< 6 µg/kg) at the end of the column experiments, owing to sufficiently high liquid-to-solid ratios (*LS*). The LS [L^3^/M] denotes the ratio of accumulated leachate volume to the dry soil mass. In the lysimeter experiment, residual soil concentrations of PFOS were higher. The predominant reason for this is incomplete leaching due to sorption. Throughout the vertical profile of the soil layer, residual concentrations ranged from 59 µg/kg PFOS at the top layer to 230 µg/kg PFOS at the bottom layer (Table S2).

**Fig. 1:**
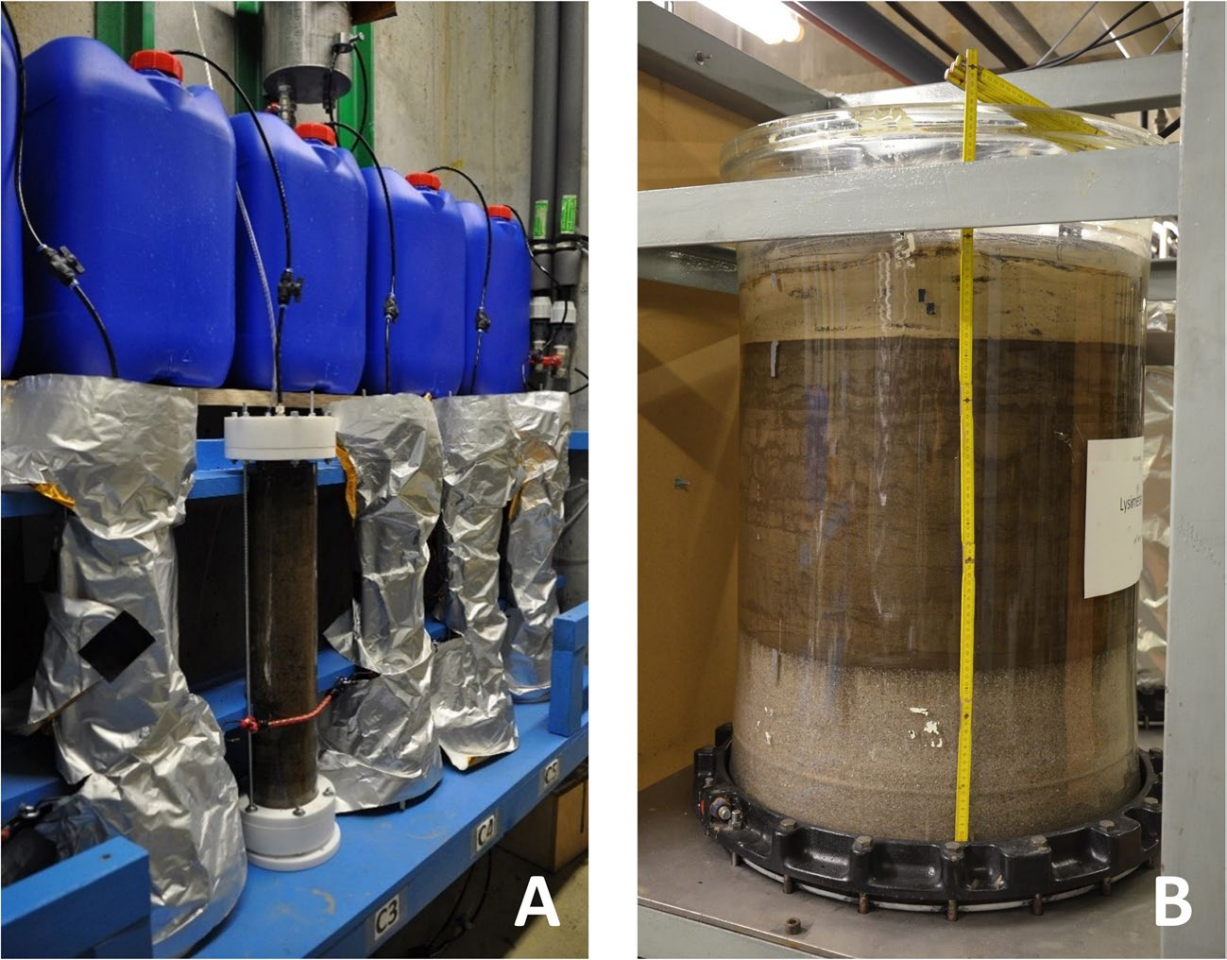
Column (**A**) and lysimeter (**B**) experiments

The experimental methods are described in detail in Bierbaum et al. (2023). Glass columns used in the saturated column experiments had a diameter of 9 cm and a length of 55 cm (Fig. 1A). Quartz sand was used at the inlet and outlet, with a thickness of 7–8 cm, to arrange uniform flow. The columns were packed with approximately 4 kg of soil material under water-saturated conditions. Four column experiments (Col1, Col2, Col3, Col4) were conducted using a peristaltic pump (demineralized, degassed water), each with a different flow rate and contact time (Col1: ≈ 5 h, Col2: ≈ 11 h, Col3: ≈ 18 h, Col4: ≈ 48 h; Table S3) to examine non-equilibrium leaching effects. Generally, the operating times, lasting up to 161 d, were longer than those usually applied in column leaching tests.

The lysimeter experiment was conducted using a glass column with a diameter of 60 cm on a perforated stainless-steel board (Fig. 1B). A quartz sand filter layer (1–2.5 mm) was established at the bottom with a length of 30 cm. The contaminated soil (186 kg, Table S3) was packed under saturated conditions, including the sand layer. Drip irrigation was adjusted to 0.9–1.1 L/d which corresponds to approximately three times the groundwater recharge in the region of the contaminated site (Baden-Baden/Rastatt, Germany).

At the start of the lysimeter experiment, a valve below the initially saturated sand layer was opened, initializing drainage of both soil and sand layers. Consequently, the outflow was high (approximately 10 L in 10 s) in the initial phase due to low water-holding capacity of the sand. After 2 d, a relatively constant outflow rate was reached at approximately 0.96 L/dy. The varying color and opacity of leachate samples indicate leaching of soil constituents such as colloids, humic acids, and other organic matter (Fig. S1). This was also observed in samples of the column experiments with a high opacity in early samples and a decreasing trend suggesting decreasing content of soil and organic matter. Furthermore, the downward migration of soil matter was visible in the top part (5 cm) of the sand layer. Additional analyses to quantify or characterize the leaching of colloids such as particle size analysis (Borthakur et al. 2021) were not performed in this study.

It is proposed that colloid-facilitated transport is causing an early, accelerated leaching (“premature leaching”) of PFOA and PFOS. Due to packing under saturated conditions, the soil structure is disturbed at the beginning of the experiment. Furthermore, rather high flow occurs at the initial drainage before a quasi-constant flow regime is reached. Thus, fine soil particles (colloids) may have been mobilized at the beginning of the experiment. Consequently, PFAS bound to colloids may have leached faster compared to transport without colloids. Faster leaching due to macropores or fractures is supposed to be unlikely due to the completely homogeneous soil structure and unsaturated conditions at *t* > 1 d.

The pressure–saturation relationship and van Genuchten parameters (Table S4) (van Genuchten 1980) were obtained by measuring soil moisture tension curves (HYPROP, Meter Group AG) with a 250-mL steel cylinder. The van Genuchten parameters were used in the continuum model of the lysimeter. At the end of the lysimeter experiment, a saturation of 0.67 was measured in the center of the soil layer.

### Continuum model

One-dimensional numerical simulations of water flow and solute transport were conducted using the program HYDRUS (HYDRUS-1D and HYDRUS 5; Šimůnek et al. 2008; Silva et al. 2020). Given that this is a continuum model, we subsequently refer to this as the *CM*.

The soil and sand layers were configured according to the experimental setup. Each layer was treated as fully homogeneous without preferential flow paths, meaning that water flow was uniform. The column experiments were simulated under saturated conditions. Although the water flow was relatively constant, it was set to correspond to the leachate volumes of each sample interval, which was adjusted by the respective pressure heads at the inlet and outlet. The lysimeter was initially saturated, as in the experimental setup. Free drainage was set as a boundary condition at the bottom, while an infiltration rate at the top corresponded to the outflow observed in the experiment. Water flow was calculated using the Richards equation (Richards 1931).

PFAS transport was derived based on the advection–dispersion equation. For the most basic model, we used an equilibrium sorption model (*equ*) where partitioning between liquid and solid phases is governed by Freundlich isotherms (Freundlich 1907) with

1 $$s={ K}_{d}{c}^{\beta },$$

where *s* [M/M] is the sorbed concentration, *K*_d_ [L^3β^/M^β^] is the Freundlich partitioning coefficient, *c* [M/L^3^] is the concentration of the liquid phase, and *β* [ −] is the Freundlich exponent (*β* = 1 for linear isotherms). Linear and non-linear sorption isotherms are explored to fit to the observed leaching. We adopted the Freundlich isotherm, in line with the majority of studies (Brusseau et al. 2019a; Guelfo et al. 2020; Silva et al. 2022a) that favor it over the Langmuir isotherm (Gagliano et al. 2020) for modeling the partitioning between liquid and solid phases. Additionally, considering the relatively low PFAS concentrations (< 1 mg/L), we did not expect that the sorption capacity is reached, a feature that is addressed in the Langmuir isotherm (Sima and Jaffé 2021).

As a more advanced and the state-of-the-art approach to model non-ideal PFAS leaching (Brusseau et al. 2019a; Guelfo et al. 2020), a dual domain sorption model (two-site sorption, *2ss*) was used enabling both equilibrium sorption and rate-limited (or kinetic) sorption (van Genuchten and Wagenet 1989; Šimůnek et al. 2008). The fraction *f* defines the fraction of the sorption sites to be in equilibrium with the liquid phase with

2 $${s}_{e}=f s,$$

and

3 $$s= {s}_{e}+ {s}_{k},$$

where *s* [M/M] is the total sorbed concentration, *s*_e_ [M/M] is the sorbed concentration of the equilibrium phase, and *s*_k_ [M/M] is the sorbed concentration of the rate-limited phase.

The Freundlich isotherm was used for sorption sites under equilibrium conditions with

4 $${s}_{e}= {f K}_{d}{c}^{\beta }.$$

Sorption of the rate-limited fraction (1 − *f*) is controlled by a first-order kinetic process with

5 $$\frac{{\partial s}_{k}}{\partial t}= \alpha \left[\left(1-f\right){K}_{d}{c}^{\beta }- {s}_{k}\right],$$

where *α* [1/T] is the first-order kinetic rate.

In each simulation, the total observed mass in the respective experiment, *m*_tot_ (sum of mass in leachate, *m*_out_, and residual mass in soil), was used as the mass reference (Table S1).

An initial liquid concentration *c*_*0*_ > 0 was set, since the soil had been in contact with water for a sufficient amount of time (saturated packing) at the start of the leaching tests. The concentration of the first leachate sample served as a proxy for the initial concentration. Equation (4) directly provides *s*_e_ under equilibrium conditions; *s*_k_ was then calculated with

6 $${s}_{k}= \frac{{m}_{tot}}{{m}_{s}}- {s}_{e}-\frac{{c}_{0} {V}_{p}}{{m}_{s}},$$

where *m*_tot_ [M] is the total PFAS mass (PFOA or PFOS), *m*_s_ [M] is the soil mass in the respective experiment, and *V*_*p*_ [L^3^] is the pore volume in the soil layer. The sand layer is treated as inert material (Lv et al. 2018), meaning that sorption to sand grains is not occurring in the simulations.

The inlet boundary condition for solute transport was set to *c* = 0, the outlet boundary condition was set to *∂c/∂x* = 0.

Sorption to AWI is simulated as an instantaneous process based on a linear partitioning relationship between the liquid phase and AWI:

7 $$\mathrm\Gamma=K_{ia}c,$$

where *Г* [M/L^2^] is the concentration on AWI and *K*_ia_ [L^3^/L^2^] is the partition coefficient of solutes between liquid phase and AWI. A number of studies use a concentration-dependent *K*_ia_ and derived *K*_ia_ from the interfacial tension and using the Gibbs equation (Adamson and Gast 1967; Lyu et al. 2018; Brusseau and van Glubt 2019). At low concentrations, the interfacial tension remains relatively constant, and measurement of changes is challenging. Therefore, at low concentrations, the Gibbs equation might not be applicable (Vecitis et al. 2008; Schaefer et al. 2019). Therefore, instead of using the Gibbs equation, we assume *K*_ia_ to be constant for concentrations below 1 mg/L. No competition between individual PFAS was simulated as this was assumed to be negligible at concentrations < 1 mg/L (Silva et al. 2021).

The magnitude of adsorption to AWI greatly depends on the area of the AWI, *A*_ia_. Various methods exist to calculate the AWI area as a function of the water content *θ* [L^3^/L^3^] (Silva et al. 2022b). We used the relation proposed by Bradford et al. (2015) which is implemented in the HYDRUS software (Bradford et al. 2015; Silva et al. 2020) with

8 $${A}_{ia}= \frac{{\rho }_{w}g}{\gamma }{\int }_{\theta }^{{\theta }_{s}}h\left(\theta \right) d\theta .$$

Hence, the AWI area increases with decreasing saturation. Higher AWI area enables a higher total mass on AWI at a given concentration in the liquid phase. The resulting relation between saturation and *A*_ia_ for the soil and sand layers in the lysimeter experiment is shown in Fig. S2.

Retardation coefficients using linear sorption isotherms are determined with

9 $$R=1+ \frac{{K}_{d} {\rho }_{b}}{\theta }+\frac{{K}_{ia} {A}_{ia}}{\theta }=1+{R}_{s}+ {R}_{ia}$$

with the retardation coefficients for solid phase adsorption *R*_*s*_ and for the AWI adsorption *R*_ia_ (Brusseau et al. 2019b).

We followed an iterative process of model parameterization through simulation and comparison to observed data. After discretization of the parameter space, a grid search method was employed, running multiple simulations with varying parameter values within a plausible range. The simulated data were then compared to the observed data through visualization and analysis to identify parameter values that result in a good fit. In the next step, additional simulations with a finer parameter range were conducted to further refine the parameter values. Finally, the best parameter set was identified with a focus on the most relevant characteristics, i.e., concentration peaks and long tailing. Additionally, the root mean squared logarithmic error (RMSLE) was used as a measure for the model fit with

10 $$RMSLE= \sqrt{\frac{1}{m} \sum\nolimits_{i=1}^{m} {\left[log\left({x}_{i}+1\right)-\mathrm{log}\left({y}_{i}+1\right)\right]}^{2} },$$

where *x* is the predicted value, *y* is the observed value, and *m* is the total number of observations.

In the column experiments with varying flow rates, the column experiment Col2 (with a contact time of 11 h) was primarily used to estimate the fitting parameters (*K*_d_, *β*, *f*, *α*). Note that *f* = 1 and *α* = 0 1/d if only equilibrium sorption between liquid and solid phases is considered. These transport parameters were then applied to simulate the other column experiments with varying flow rates. Due to the variation in total masses observed across the individual experiments, the initial concentrations (*c*, *s*_e_, *s*_k_) also varied accordingly (Table S5). In simulations using equilibrium sorption (*f* = 1), the *K*_d_ values were determined by adjusting the initial liquid concentration and aligning the *K*_d_ value with the total mass in the respective experiment. These *K*_d_ values were used as an approximation for the *2ss* model. In simulations of the lysimeter experiment, *K*_ia_ was introduced as another fitting parameter. Thereby, for *β*, the value previously estimated in the column experiments was used.

### Random walk particle tracking model

A one-dimensional random walk particle tracking (PT) model (Ahlstrom et al. 1977; Dentz and Berkowitz 2003) was developed to simulate leaching of PFAS in the lysimeter. Contrary to continuum models, random walk particle tracking does not require the numerical solution of the advection–dispersion equation (Dentz and Berkowitz 2003; Henri and Diamantopoulos 2022). Instead, the motion of individual particles or solutes is directly simulated using advective and dispersive motion components, combined with stochastic processes to model mass transfer between mobile and immobile phases (Henri and Fernàndez-Garcia 2015; Hansen and Berkowitz 2020b). Each simulated particle represents a defined unit of PFAS and moves independently from other particles but is affected by advection, diffusion, and sorption. In each time step Δ*t*, which was constant in our model, the position of particles (*x*_*n*_) in a mobile state was updated according to

11 $${x}_{n+1}= {x}_{n}+v\left({x}_{n},t\right)\Delta t+ {\Delta }_{x,n}^{D} ,$$

where *x*_n+1_ is the updated position, *v(x*_n_,*t)* is the flow velocity, and ${\Delta }_{x,n}^{D}$ is the dispersive term with

12 $${\Delta }_{x,n}^{D}=N(\mathrm{0,2}{\alpha }_{L}v\left({x}_{n},t\right)\Delta t ,$$

which is a random number sampled from a normal distribution with a mean *µ* = 0 and the variance *σ*^2^ = $2{\alpha }_{L}v({x}_{n},t)\Delta t$ (see also Hansen and Berkowitz 2020a).

The flow velocity was obtained by

13 $$v\left({x}_{n},t\right)= \frac{q\left(t\right)}{n\left({x}_{n}\right){s}_{w}\left({x}_{n}\right)} ,$$

where *q(t)* [L/T] is the observed discharge (leachate volumes divided by sample interval), *n*(*x*_n_) [ −] is the porosity, and *s*_*w*_(*x*_n_) [ −] is the saturation. For *t* < 0.8 d, saturation values of 0.67 and 0.30 were set in soil and sand layer, respectively. For *t* > 0.8 d, the saturation was set constant with 0.57 in the soil layer and 0.19 in the sand layer. These values were derived from simulation results of the CM, the latter were the mean of the saturation values in each layer at the end of the simulation at *t* = 888 d.

The observed premature leaching was modeled as a separate process employing a retardation in the advective transport term. Colloid-facilitated transport is simulated with

14 $${v}_{coll}\left({x}_{n},t\right)= \frac{q\left(t\right)}{n\left({x}_{n}\right) {s}_{w}\left({x}_{n}\right) {R}_{coll,i}} ,$$

where *R*_coll_ is the retardation coefficient for the colloids and subscript *i* denotes the soil or sand layer. We did not simulate the attachment or detachment of PFAS to/from colloids. Instead, we used retardation coefficients as a fitting parameter to characterize the observed premature leaching. Adding more complexity to the model by including ad- and desorption rates of PFAS to/from colloids would require knowledge about the time- and position-dependent colloid density, as well as possible differences in sorption rates due to colloid-specific properties that may differ from the mean soil properties.

Alternatively, if colloid-facilitated transport was not considered, we used a substance-specific retardation coefficient to model the premature leaching of PFOA and PFOS independently, resulting in

15 $${v}_{pre,l}\left({x}_{n},t\right)= \frac{q\left(t\right)}{n\left({x}_{n}\right) {s}_{w}\left({x}_{n}\right) {R}_{pre,l,i}} ,$$

where *l* denotes the individual PFAS. Like the colloid-facilitated leaching, we did not account for any adsorption or desorption for this particle fraction.

Each particle in the simulation was assigned a specific PFAS mass based on the total number of particles and the overall PFAS mass in the experiment. A large number (> 100,000) of particles was used. Initially, the particles were evenly distributed throughout the soil profile. Each particle was given an initial state, which could be “dissolved,” “sorbed to soil,” or “sorbed to colloids.” The relative composition of the initial states reflected the initial partitioning and the initial concentrations in the liquid phase, on the solid phase, and on colloids. Due to the initially saturated conditions in the experiment, the state “sorbed to AWI” did not exist initially. PFAS was absent in the sand layer.

The transition between mobile (dissolved) and immobile (sorbed to soil, sorbed to AWI) phases was governed by adsorption (*λ*) and desorption (*µ*) probabilities (Hansen and Berkowitz 2020b), which represented the first-order rates of state transitions. In practical terms, this was implemented by generating a random number from a uniform distribution between 0 and 1. If the random number was below the respective transition rate (s^−1^), the state of the particle would change accordingly. These ad- and desorption rates were the primary fitting parameters in the PT to simulate the steady-state leaching.

The physical properties of the solid phase and AWI were not incorporated into the model. The AWI remained constant throughout the entire domain and was not influenced by changes in saturation.

Retardation coefficients were determined (Hansen and Vesselinov 2018):

16 $$R=1+ \frac{{\lambda }_{s}}{{\mu }_{s}} + \frac{{\lambda }_{ai}}{{\mu }_{ai}}=1+{R}_{s}+ {R}_{ia}.$$

## Results and discussion

### Simulation of leaching under saturated conditions

The leaching characteristics of PFOA and PFOS in the column experiments have been described briefly in Bierbaum et al. (2023) but are summarized here for completeness. Both PFOA and PFOS exhibited early concentration peaks followed by extended tailing during the leaching process. Variability in PFOA and PFOS concentrations in the initial leachates of individual experiments can be primarily attributed to differences in *LS* and the varying volumes of cumulative leachate samples. The observed concentration ranges extended across multiple orders of magnitude (52 µg/L to ≈ 100 ng/L). Peak concentrations of PFOA were observed to range from 13 to 52 µg/L, those of PFOS from 26 to 32 µg/L. The major leaching of PFOA occurred faster than that of PFOS, attributable to PFOA’s lower sorption affinity (Higgins and Luthy 2006; Guelfo and Higgins 2013; Xiao et al. 2017; Fabregat-Palau et al. 2021). However, both PFOA and PFOS concentrations eventually stabilized at similar long-term levels of approximately 100 ng/L.

As the most elementary model in this study, PFAS leaching was simulated applying equilibrium sorption conditions (*f* = 1). By doing so, the early breakthrough was reproduced for all experiments with different flow rates, but the extensive tailing is not captured (Fig. S3). *K*_d_ values of 0.74 cm^3^/g and 3.3 cm^3β^/g^β^ were found for PFOA and PFOS (Table 2), respectively, to yield a good fit to all column experiments with different flow rates. This reflects the higher hydrophobic interactions of PFOS and more distinct retardation. This is in accordance to several other studies (e.g., Guelfo et al. 2020; van Glubt et al. 2021; Schaefer et al. 2022b). Note that comparing *K*_d_ values to studies using different soil material is difficult since sorption strongly depends on soil properties like the content of organic matter and iron oxides.

**Table 2:** CM parameter values for the equilibrium (*equ*) and two-site sorption (*2ss*) models used in the simulations of saturated column experiments

	Sorption model	*f* [ −]	*K*_d_ (cm^3β^/g^β^)	*β* [ −]	*α* (1/d)
PFOA	*equ*	1^+^	0.74	1.0	–
PFOA	*2ss*	0.93	1.0	1.0	0.01
PFOS	*equ*	1^+^	3.3	0.9	–
PFOS	*2ss*	0.93	4.5	0.98	0.005

+: indicates the parameters which are predetermined by the model choice

The fitting of sorption parameters on the observed leaching of PFOA has a higher uncertainty due to the low sampling frequency in the early part of the experiments (*LS* < 2 L/kg). The peak concentration of PFOA in Col1 suggests a lower *K*_d_ value (and consequently a higher initial concentration in the liquid phase). However, this would affect the fit for the other column experiments negatively. Hence, the here presented *K*_d_ values represent a reasonable average fit to all experiments.

The *K*_d_ values in this study are meaningfully lower than the *K*_d_ values resulting from the calculation based on *f*_oc_ and the organic carbon–water partition coefficient (*K*_oc_) which is conventionally used with *K*_d_ = *f*_oc_ × *K*_oc_. In the studied soil (*f*_oc_ = 0.018), with a *K*_oc_ of 350 L/kg for PFOA and 1600 L/kg for PFOS (Nguyen et al. 2020), this would result in *K*_d_ values of 6.3 cm^3^/g for PFOA and 28.8 cm^3^/g for PFOS. While our experiments suggest one order of magnitude smaller *K*_d_ values, the ratio of the fitted *K*_d_ values for PFOA and PFOS agrees with the ratio of the *K*_d_ values based on *f*_oc_ and *K*_oc_. Overall, derivation of *K*_d_ values solely based on single soil properties has been found to be an oversimplified approach and can only offer a rough estimation (Higgins and Luthy 2006; Milinovic et al. 2015; Li et al. 2018; Fabregat-Palau et al. 2021).

While a linear sorption isotherm was sufficient to simulate the equilibrium fraction of PFOA, a better fit was achieved for PFOS when *β* < 1 was used. A higher value for *K*_d_ instead of using non-linear isotherms cannot be applied while maintaining mass balance and fitting to the early leachate concentrations. In literature (Brusseau et al. 2019a; Guelfo et al. 2020), both linear and non-linear isotherms were used to fit observed breakthrough curves. Brusseau et al. (2019a) determined *β* values of 0.77 and 0.81 for PFOS; Guelfo and Higgins (2013) reported values of 0.7–1.1 for various PFAAs. Nonetheless, the extensive tailing observed in the present study cannot be reproduced by using non-linear isotherms alone indicating the influence of rate-limited sorption. The sorption parameters determined using the equilibrium model were considered a reasonable approximation for the subsequent simulations using the *2ss* model.

The difference between the *equ* model simulations and the observed long tailing suggests the dominance of another sorption process at higher *LS*. The transition from sorption controlled by equilibrium conditions to kinetically controlled sorption can be approximated by identifying where the equilibrium model and observed leaching characteristics diverge. Using this simple approach, leaching of PFOA becomes kinetically controlled at *LS* ≈ 4 L/kg (Fig. S4A) and the leaching of PFOS at *LS* ≈ 20 L/kg (Fig. S4B). The transition from leaching controlled by equilibrium sorption to rate-limited sorption is less distinct for PFOS than for PFOA, as the change in slope is less pronounced.

**Fig. 2:**
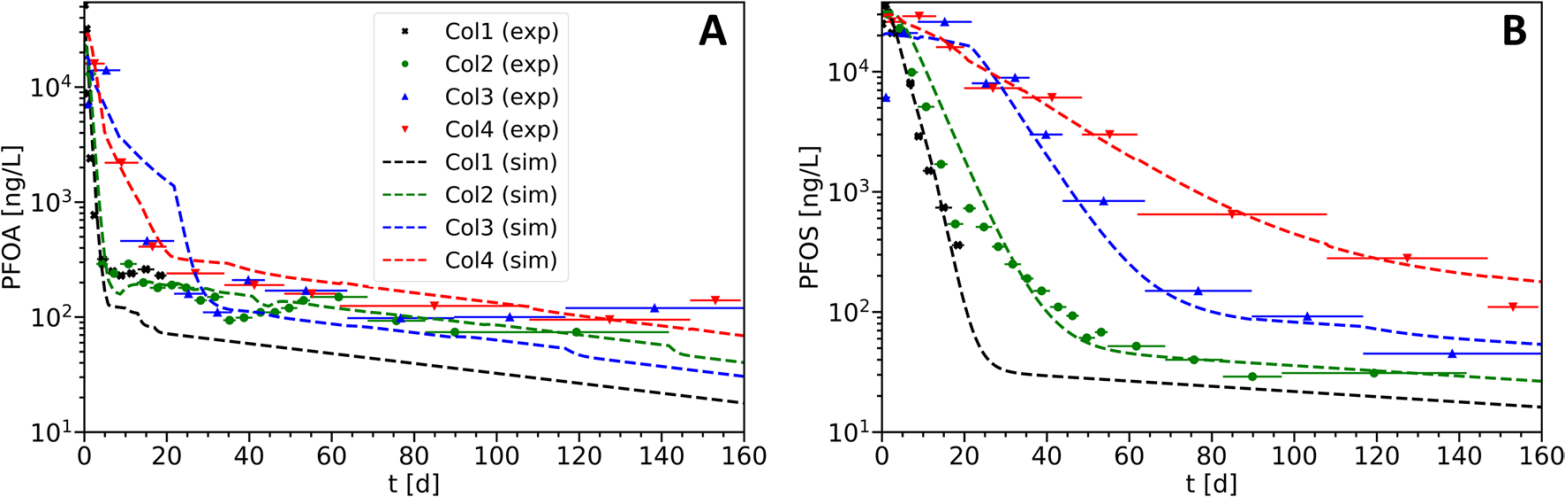
Observed (markers with horizontal bars) and *2ss* model–simulated (dashed lines) leachate concentrations of PFOA (**A**) and PFOS (**B**) in the saturated column experiments with varying flow rates. Horizontal bars in the observed data represent their corresponding sampling intervals. Different colors and markers denote individual experiments

The *2ss* model was found to reproduce the observed leaching characteristics of PFOA and PFOS in saturated experiments with varying flow rates (Fig. 2). While equilibrium sorption is dominant during early leaching stages, rate-limited sorption controls long-term leaching. The *K*_d_ values obtained from fitting the equilibrium sorption model were used as a guide, with minor variations tested. The fitted *K*_d_ values for the *2ss* model were slightly higher than those used in the equilibrium model (Table 2), likely due to fractionation between equilibrium and kinetic phases and the method used to adjust concentration on the rate-limited sites (Eq. (6)). Increasing *K*_d_ at a given fraction results in a higher initial sorbed concentration on the equilibrium phase. The initial concentrations of the rate-limited sorption sites vary among the individual experiments. This is mainly a result of varying observed masses and initial liquid concentrations in the individual column experiments. Above all, the mass balance was respected as presented in Eq. (6).

The *2ss* model suggests a higher variability in the long-term leachate concentrations of PFOA among the different column experiments compared to the observed values. The observed leachate concentrations at *t* = 100 d range from approximately 70 to 150 ng/L, while the simulated concentrations range from 30 to 200 ng/L. The simulations slightly overestimate the leachate concentrations in the experiments with low flow rates (Col4) and underestimate them in the experiments with high flow rates (Col1). Thus, varying the parameterization of the rate-limited sorption would not improve the overall fit for all experiments.

While the overall fit to the data is generally good, the long-term PFOS concentrations at lower flow rates (Col3, Col4) are slightly overestimated, which is in accordance with the observations for PFOA. For PFOS, rate-limited sorption is dominating the leaching characteristics at higher *LS* compared to PFOA due to PFOSs generally higher sorption affinity. As equilibrium sorption of PFOS is still significant at the conclusion of some experiments, the examination of rate-limited leaching is more uncertain due to limited data. Therefore, the leaching data of the experiments Col2, Col3, and Col4 were primarily used to fit rate-limited sorption parameters. Col1 was stopped before transition to the supposed kinetically controlled leaching. Despite the long experimental times and high *LS* of the dataset used in this study, an even more extended time series would be beneficial for fitting sorption parameters for the long tailing. Nevertheless, the parameters determined are considered reasonable.

A sensitivity analysis was carried out to explore the impacts of varying *α* and *f* on the model’s performance (Fig. S5 and S6). It was found that modifying *α* by a certain factor led to a proportional change in long-term leaching. Increasing *α* emphasized its role in comparison to equilibrium sorption, but it also caused the depletion to happen more rapidly. In the case of *f*, a lower value led to higher long-term leachate concentrations, as it caused a higher initial *s*_k_ and consequently enhanced mass transfer. Although the fit to the data worsened when parameters were altered individually, the presented parameterization may not be unique; different combinations of values for *α* and *f* could potentially be valid; however, the here presented parameterization provided the most satisfactory results in our findings. Using a fraction value lower than 0.8 was found to be unsuitable for PFOA and PFOS, as it would underrepresent equilibrium sorption and cause the steep concentration decline to occur more rapidly than the data suggests.

The fitted model parameters suggest less dominant rate-limited sorption for PFOS compared to PFOA. The ratio of initial concentrations on the kinetic sites to the initial concentrations on the equilibrium sites is lower for PFOS (Table S5). Concomitantly, *α* used for PFOS is half the value used for PFOA (Table 2), i.e., the ad- and desorption rates are lower for PFOS. This is consistent with Nguyen et al. (2022) who found that lower values for *α* and *f* are applicable for more hydrophobic PFAAs and suggested that hydrophobic interactions rather than electrostatic interactions cause rate-limited leaching. PFOS is more hydrophobic compared to PFOA due to the additional CF_2_ attached but has similar electrostatic properties (Zeng et al. 2020). Electrostatic interactions are widely considered to be instantaneous and faster than hydrophobic interactions. Possibly, the rate limitations of PFOA and PFOS are affected by variation in diffusion properties associated with their respective functional groups (Guelfo et al. 2020).

The suggested *f* and *α* (Table 2) are consistent with reported values in the literature. Guelfo et al. (2020) reported rate values ranging from 10^−3^ to 10^−8^ h^−1^. Silva et al. (2022a) used *f* = 0.84 and *α* = 0.003 d^−1^ for long-term simulations of several individual PFAS where non-ideal transport was observed. Compared to this study, this relates to a lower fraction and lower *α*—meaning, more sorption sites were kinetically controlled, while the sorption rates were lower. When comparing *f* and *α* among different studies, one must be aware of their dependency: Similar leachate concentrations may result using a higher *f*, i.e., less sorption sites are rate limited, in combination with a higher *α*. In contaminated soils, the depletion of the mass initially sorbed on kinetic sorption sites occurs earlier using a higher *α*.

The residual concentrations in the *2ss* model fall within the range of the experimental data. Measured residual soil concentrations of PFOA and PFOS at the end of individual experiments vary between 1.8–3.9 µg/kg and 3–5 µg/kg (Table S1), respectively, with the highest concentrations found in experiments reaching the lowest *LS* values. Since rate-limited sorption governs long-term leaching, these values can be compared to the residual concentration of kinetic sites at corresponding times. The simulated residual concentrations display a slightly higher variability across different experiments (Table S6). Compared to experimental data, both PFOA and PFOS exhibit higher residual concentrations in experiments with higher flow rates (Col1: 8.3 µg/kg versus 3.9 µg/kg, Col2: 2.6 µg/kg versus 1.8 µg/kg). Moreover, lower residual PFOA concentrations were observed in experiments with lower flow rates (Col3: 1.1 µg/kg versus 3 µg/kg, Col4: 1 µg/kg versus 3.2 µg/kg), while the opposite trend was observed for PFOS (Col4: 4.8 µg/kg versus 3.1 µg/kg). It is important to note that residual concentrations in the kinetic phase are strongly dependent on the initial *s*_k_ (Table S5). As different total masses lead to varying initial *s*_k_ (Eq. (6)), drawing conclusions becomes difficult. Nevertheless, by trend, the experimental data show lower variability in residual concentrations across experiments with varying flow rates and operation times. The largest deviation of the model occurs in the experiment with the lowest reached *LS* and the highest flow rate, Col1. With a residual concentration of 3.9 µg/kg PFOA (at *LS* ≈ 19 L/kg) and a presumed leaching rate of 0.25 µg/kg/d (linear trend of Col1), all PFOA would be completely leached after an additional 16 d which was not observed in the other experiments that reached *LS* > 40 L/kg.

The contribution of precursor transformation to the observed long tailing in PFAS leaching remains uncertain. Incomplete mass balances, showing 80 to 90% recovery for PFOA and PFOS, suggest that transformations are not significant in the column experiments (Bierbaum et al. 2023). Röhler et al. (2021) compared leaching data from column experiments to field data and found that long-term leaching was not sufficiently represented in the column experiments due to relatively short experimental times (approximately 2 weeks). Maizel et al. (2021) conducted column experiments with biotic and sterilized soil and did not observe differences, concluding that transformation was negligible. In the present study, column experiments reached higher *LS* and had longer operating times (up to 160 d) compared to the mentioned studies. Therefore, transformation possibly becomes relevant towards the end of the experiments. This might be reflected in the discrepancy between the predicted monotonously decreasing PFOA concentrations and the rather constant leaching observed in the experiment for *t* > 100 d (Fig. 2). Nevertheless, we demonstrated that the observed leaching characteristics can be reproduced to a satisfactory degree solely considering sorption processes with the *2ss* model. Precursor transformation may be more relevant in the lysimeter experiments due to aerobic conditions (Zhang et al. 2013, 2016; Yin et al. 2018; Yi et al. 2018) and longer operating times, which is further discussed in the “Simulation of leaching under variably saturated conditions” section.

### Simulation of leaching under variably saturated conditions

#### Leaching characteristics in the lysimeter experiment

The leaching characteristics observed in the variably saturated experiments differ significantly to those observed in the saturated column experiments, with the differences being more pronounced for PFOS than for PFOA. For PFOS, two distinct leaching events were observed: an early breakthrough of a minor PFOS fraction (approximately 3% of *m*_tot_ = 205 µg/kg) at *t* < 200 d (premature leaching), followed by sustained steady-state leaching up to ≈ 200 d (Fig. S1). The premature leaching event is characterized by a local concentration maximum of 11 µg/L at *t* < 7 d, followed by a decrease in concentrations to a minimum of 1 µg/L at 176 d. Subsequently, leachate concentrations increase again, reaching 21 µg/L until the end of the experiment, with some fluctuations. Very similar leaching characteristics were observed for PFNA and PFDA.

Considering the steady-state leaching at *t* > 200 d, the leaching of PFOS is much more retarded relative to the saturated column experiments where instant breakthrough is observed. Instead, increasing concentration at *t* > 200 d is observed. Most likely, this retardation is due to sorption to AWI, especially within the sand layer. It is supposed that PFOS accumulates on the free sorption sites on AWI causing a drastic delay in the breakthrough. In the sand layer, quartz sand was used and washed before the experiment to flush out fine particles. Consequently, the specific surface area of the quartz sand is small, and sorption to the sand particles is assumed to be negligible. Thus, adsorption to AWI is assumed to be the only relevant sorption mechanism in the sand layer. As a separate process for a minor PFOS fraction, colloid-facilitated transport is proposed for the premature leaching event, which is addressed below.

For PFOA, the pattern is less clear and less pronounced than for PFOS. Nevertheless, the leaching curve also exhibits two peaks with concentration maxima at *t* = 30 d with 34 µg/L and at *t* = 204 d with 23 µg/kg (Fig. S1). Subsequently, PFOA concentrations decrease to rather constant outflow concentrations < 1 µg/L. It is known that PFOA is less surface active than PFOS which results in a lower sorption affinity to air–water interfaces. Thus, the retardation due to sorption at AWI is lower for PFOA resulting in a superposition of the two proposed leaching processes (premature leaching and steady-state leaching).

#### CM simulations

The CM showed that saturation remained relatively constant over time after the initial drainage, with saturation ranging from 0.49 to 0.55 in the center of the soil layer for *t* > 7 d. At the end of the experiment (*t* = 888 d), saturation varied across the soil profile ranging from 0.41 at the top to 0.83 at the transition to the sand layer, with a mean of 0.57. In the sand layer, saturation was relatively constant over the vertical profile at 0.19. This corresponded to mean AWI areas of *A*_ia_ of 80 cm^−1^ in the soil layer and 43 cm^−1^ in the sand layer, which fell within the reported range of *A*_ia_ values (Lyu et al. 2018; Silva et al. 2019; Abraham et al. 2022).

**Fig. 3:**
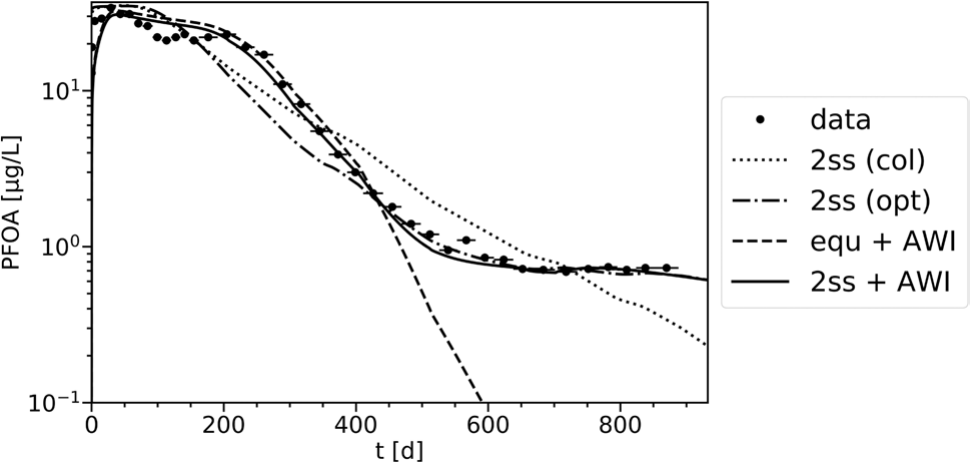
Observed (● with horizontal bars) and simulated PFOA leachate concentrations in the lysimeter experiment. The series *2ss* (*col*) (dotted line) used parameter values previously employed for the column experiments (Table 2). Based on this, in the *2ss* (*opt*) series (dash-dotted line), *α* was decreased to 0.0008 d^−1^. Both series *equ* + *AWI* (dashed line) and *2ss* + *AWI* (solid line) simulations incorporated sorption to AWI. Refer to Table 3 for parameter values

The *2ss* model parameters used for the column experiments (Table 2) were not suitable for simulating the leaching observed in the variably saturated lysimeter experiments (Fig. 3, series *2ss* (*col*)). The predicted PFOA leaching has a lower slope compared to the observed leaching, and the overall characteristic is not adequately captured. It is possible that the misfit at *t* < 400 d arose from the premature leaching and sorption to AWI, which is further examined below. Particularly, the predicted rate-limited PFOA leaching significantly differed from the observed long-term leaching for *t* > 600 d. With the given parameterization, the depletion of PFOA mass sorbed on kinetic sites occurs relatively early (*t* < 700 d), leading to lower long-term leachate concentrations. However, leaching proceeds for *t* > 700 d at an almost constant concentration level in the experiment. Possibly, this hints at the importance of transformation for the long-term leaching.

If the long-term leaching was still dominated by sorption processes and the contribution of precursor transformation is negligible, the *2ss* model suggests that rate-limited leaching is significantly lower under variably saturated conditions. The lower mass transfer rates from kinetic sites may be due to a higher proportion of sorption sites in equilibrium as a result of lower flow rates. To improve the model fit to the observed long-term leaching (Fig. 3, series *2ss opt*), *α* was decreased by a factor of 13 (from 0.01 d^−1^ to 0.0008 d^−1^, Table 3); concomitantly, a lower initial *s*_k_ value (4.7 µg/kg, Table S5) was used compared to the column experiments. This observation implies that parameters obtained from saturated conditions with higher flow velocities may not be directly transferable to variably saturated and realistic field-scale conditions. Thus, accurately fitting rate-limited leaching parameters for long-term simulations requires a comprehensive database with diverse conditions. Furthermore, these findings suggest that rate-limited leaching may be less significant under field conditions compared to saturated column experiments, emphasizing the importance of lysimeter experiments.

**Table 3:** CM parameter values for the equilibrium (*equ*) and two-site sorption (*2ss*) models used in the simulations of the lysimeter experiments

PFAS	Sorption model	*f* [ −]	*K*_d_ (cm^3β^/g^β^)	*β* [ −]	*α* (1/d)	*K*_ia_ (cm)
PFOA	*2ss*	0.93	1.0	1.0	0.0008	–
PFOA	*equ* + *AWI*	1^+^	1.0	1.0	–	0.007
PFOA	*2ss* + *AWI*	0.93	1.0	1.0	0.001	0.007
PFOS	*equ* + *AWI*	1^+^	4.5	0.98	–	0.15

Note that values for f and β were determined during preliminary simulations of the column experiments (Table 2); Kd, α, and Kia were estimated

+: indicates the parameters which are predetermined by the model choice

The *2ss* model simulations for PFOA (Fig. 3, series *2ss col*, and *2ss opt*) generally show faster leaching and an earlier decrease in concentration compared to the observed data. To improve the model fit, equilibrium sorption to the solid matrix and sorption to AWI following a linear Freundlich isotherm (*K*_ia_ = 0.007 cm) were included, and the model was fitted to the data at 200 d < *t* < 450 d (Fig. 3, series *equ* + *AWI*). With the 2ss model including partly rate-limited leaching with *f* = 0.93 and *α* = 0.001 d^−1^ (Table 3), the long-term leaching (Fig. 3, 2*ss* + *AWI*) is reproduced. Both simulations demonstrate that the AWI may serve as an additional sorption medium for PFOA. Nevertheless, the model did not reproduce the two local concentration maxima observed in the data (more pronounced at a linear concentration axis, Fig. S1). Further investigations on this issue using PT are described below.

The precursor content is high, and a number of observations by Bierbaum et al. (2023) indicate that the observed long-term leaching is not only a sorption process but may be influenced by transformation of precursors to PFAAs. Bierbaum et al. (2023) mainly discuss possible transformation to PFBA where they observed higher leached masses in the lysimeter experiments with longer operating times compared to the column experiments and the original soil content. This is also true for PFOA and PFOS: In the lysimeter experiment, 46.6 µg/kg PFOA and 205 µg/kg PFOS were observed, while 45.3 µg/kg PFOA and 186 µg/kg PFOS were the average soil content. Contrarily, masses observed in the column experiments were mostly below the measured soil content with 28.1–48.6 µg/kg PFOA and 108.2–174.8 µg/kg PFOS (Table S1).

As an alternative to the *2ss* models considering equilibrium and rate-limited sorption, the observed long-term leaching of PFOA can be replicated using equilibrium sorption and a stationary source term (Fig. S7). This was examined by adding a solute (100 µg/kg) with a high sorption affinity (*K*_d_ = 10,000 L/kg) and applying a transformation rate of 3∙10^−5^ d^−1^. Using this approach, potential precursors are represented by a bulk source term, with a direct conversion to PFOA. It is important to note that this is a drastic simplification of the actual situation, where various precursors, some of which are unknown, are present and transformation occurs through multiple intermediate products with varying transformation rates (Lee et al. 2014; Liu and Liu 2016; Weidemann et al. 2022). Nonetheless, this demonstrates that the observed long-term leaching may result from transformation of precursors.

Weidemann et al. (2022) investigated transformation of 8:2 diPAP which is an important precursor in the soil of this study. With the reported half-life of 630 d, 156 µg/kg 8:2 diPAP (measured soil content) in the studied soil, and 17% of the transformation product being PFOA, 0.173 µg/kg/d PFOA would be produced. This is a much higher rate than observed in the long-term leaching of the lysimeter experiments with 0.003 µg/kg/d PFOA. Hence, it is highly plausible that the observed long-term leaching at *t* > 600 d in the lysimeter is attributable to the transformation of PFOA precursors, such as 8:2 diPAP. This is consistent with Guelfo et al. (2020), who stated that precursor transformation may be more important than rate-limited leaching at field scale, and other studies (Röhler et al. 2021; Schaefer et al. 2022a; Just et al. 2022).

**Fig. 4:**
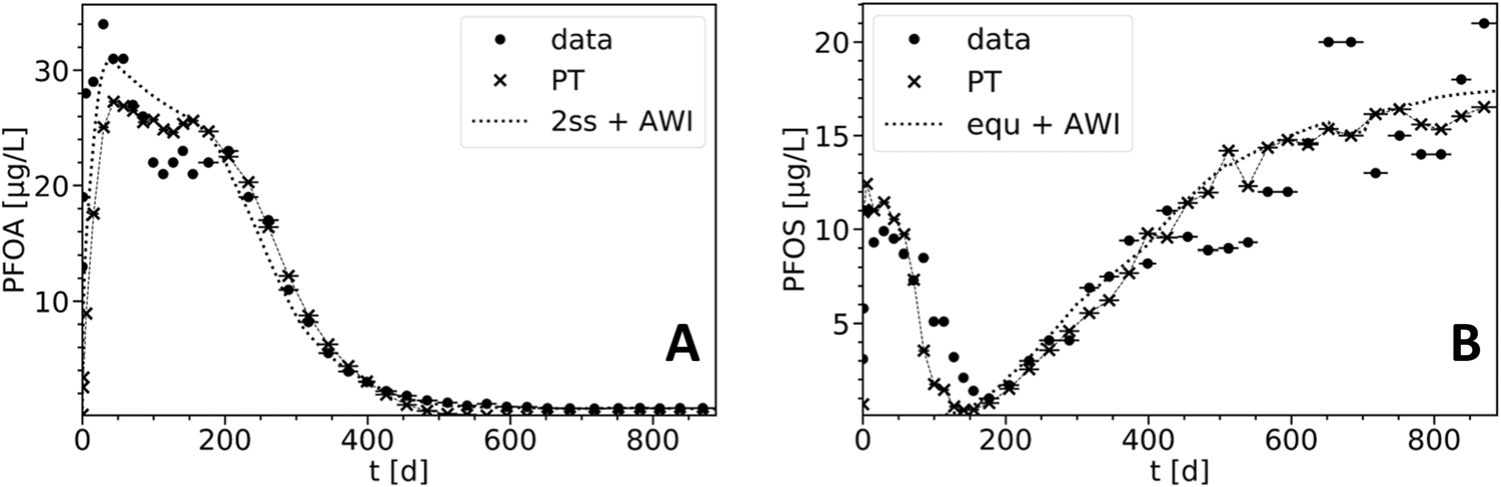
Observed (● with horizontal bars) and simulated PFOA (**A**) and PFOS (**B**) leachate concentrations in the lysimeter experiment. The CM results (**A**, *2ss* + *AWI*; **B**, *equ* + *AWI*) include sorption to AWI. The PT results additionally include partly colloid-facilitated transport using equal transport parameters for the colloid-facilitated part for both PFOA and PFOS (Eq. 14)

The simulations with the CM support that adsorption to AWI is the dominant retention mechanism for the observed leaching of PFOS. The equilibrium sorption model including sorption to AWI with a *K*_ia_ of 0.15 cm in soil and sand layers accurately reproduces the steady-state leaching of PFOS at *t* > 200 d (Fig. 4B). At *t* < 200 d, predicted outflow concentrations were negligible, suggesting significant retention of PFOS and accumulation at AWI, even at high flow velocities under initially saturated conditions. Assuming equilibrium sorption to AWI, this finding supports the hypothesis that the premature leaching of PFOS (*t* < 200 d) is a separate process caused by another sorption mechanism, such as colloid-facilitated transport.

A linear Freundlich isotherm was found to adequately model sorption to AWI for PFOS, despite Langmuir-type sorption isotherms with a maximum sorption (*Г*_max_) being more commonly used. For instance, Silva et al. (2021) used a *Г*_max_ of 5.5∙10^–6^ mol/m^2^ PFOA (0.23 µg/cm^2^) and 3.5∙10^−6^ mol/m^2^ PFOS (0.14 µg/cm^2^). The concentrations on AWI in this study are much lower (up to approximately 0.0025 µg/cm^2^) and fall within the linear range of proposed Langmuir isotherms. Thus, for a lot of model studies concerning concentrations < 1 mg/L, i.e., field-relevant concentration levels, linear Freundlich isotherms may be sufficient (Schaefer et al. 2019; Brusseau et al. 2021; Stults et al. 2023).

The general leaching trend of PFOS at *t* > 200 d is reasonably captured by the CM, although the concentration fluctuations are not as well represented (Fig. 4B). The largest deviation from the predicted values occurs at the high concentrations (≈ 20 µg/L) observed between 650 d < *t* < 700 d. During this period, the discharge is significantly lower (≈ 30%) compared to the overall quasi-constant flow regime. In contrast to the observed values, the simulated concentrations decrease, indicating a different response to the reduced flow. Similar fluctuating patterns were observed for PFNA and PFDA (Bierbaum et al. 2023). These measurements were taken towards the end of summer and beginning of autumn, when higher ambient temperatures may have enhanced PFAS leaching. Seasonal temperature fluctuations ranged between 16 and 24 °C, with the highest temperature recorded in August. It is worth noting that the contact time in the lysimeter is approximately 30 d, which could account for the delay between the peak in PFAS concentrations in September and the temperature peak in August. Simultaneously, warmer temperatures could increase microbial activity and biotransformation of precursors (Röhler et al. 2021). However, the relative contribution of PFOS production is assumed to be negligible compared to the leaching of initially present PFOS (with an initial soil concentration of about 186 µg/kg), suggesting that this behavior is primarily controlled by sorption/desorption processes. Given that sorption to AWI is the dominant sorption mechanism for PFOS, the temperature dependency of this process may be significant, potentially resulting in seasonal variations in mass flux to the groundwater at contaminated sites.

Our *K*_ia_ values for PFOA and PFOS fall within the range of reported values at similar concentration levels (0.01–0.1 mg/L). For PFOA, reported *K*_ia_ values range from 0.002 cm (Lyu et al. 2018) to 0.06 cm (Abraham et al. 2022), while for PFOS, they range from 0.027 cm (Brusseau et al. 2021) to 0.63 cm (Stults et al. 2023). Abraham et al. (2022) observed lower *K*_ia_ values for PFOA when other PFAAs were present compared to single-solute systems, concluding that competition between individual PFAS occurred. This competitive effect may also be present in the leaching data presented here, causing minor sorption to AWI for PFOA due to the presence of more surface-active compounds such as PFNA, PFDA, and PFOS. However, competitive sorption to AWI was not represented in the models since concentrations were considered too low to be significant.

#### PT simulations

The PT was employed to simulate the premature leaching (PFOS: *t* < 200 d) and steady-state leaching (PFOS: *t* > 200 d). The steady-state leaching results, including sorption to AWI, were consistent with the CM results (Fig. 4B). For solid phase sorption, the *K*_d_ values from the CM were used as a reference. Equations (9) and (16) were employed to convert the *K*_d_ values into ratios of *λ*_s_ and *µ*_s_ (Table 4). Unlike the CM, PT did not account for spatial variations in sorption to AWI. The CM resolved the AWI area based on a functional relation dependent on saturation and solid material—resulting in varying AWI area along the vertical profile—while the PT treated the AWI as an immobile phase which is constant over the vertical profile. The comparable results of PT and CM indicate that the simplifications made for accounting sorption to AWI were justified.

**Table 4:** State transition rates (*λ*, adsorption; *µ*, desorption; in s^−1^) of PFOA and PFOS used in PT simulations (*RMSLE*: 0.36 (PFOA) and 0.20 (PFOS))

	Soil		AWI	
	*λ*_s_	*µ*_s_	*λ*_ia_	*µ*_ia_
PFOA	0.0001	2.5 × 10^−5^	0.0001	1.82 × 10^−5^
PFOS	0.0001	5.88 × 10^−6^	0.0001	1.58 × 10^−6^

The simulation results suggest that the premature leaching for both PFOA and PFOS may be due to colloid-facilitated transport, which requires consistent transport characteristics for both contaminants (Eq. 14). The PT was fitted to the experimental data for PFOA and PFOS, using 3.1% initial partitioning to colloids and *R*_coll_ = 4 in the soil layer, and no retardation of the colloids in the sand layer. Consistent to the experimental data, the simulation results for PFOA showed two local concentration maxima (at *t* ≈ 50 d and *t* ≈ 150 d), and the premature leaching of PFOS at *t* < 200 d is reproduced. This demonstrates that colloid-facilitated transport may be relevant which is consistent with the study of Borthakur et al. (2021).

Due to the opposed relations between the PT results and the data for PFOA and PFOS, it was not possible to improve the model fit for both contaminants while keeping equal transport parameters for the colloids. The premature PFOA leaching for *t* < 100 d was underestimated and leaching at 100 d < *t* < 200 d overestimated compared to the experimental data (Fig. 4A). On the other hand, the simulation results for PFOS overestimated the premature leaching at *t* < 75 d and underestimated it at *t* > 75 d. The observed tailing of the premature leaching of PFOS was more enhanced compared to the simulation results. Due to the higher sorption affinity of PFOS, expectedly, colloid-facilitated transport is more significant for PFOS. Contrarily, the PT model underestimates the early PFOA leaching suggesting a more significant colloid-facilitated leaching for PFOA. While these results show that colloid-facilitated transport may be significant, they also indicate that another process may be relevant for both PFOA and PFOS which is further examined below.

**Fig. 5:**
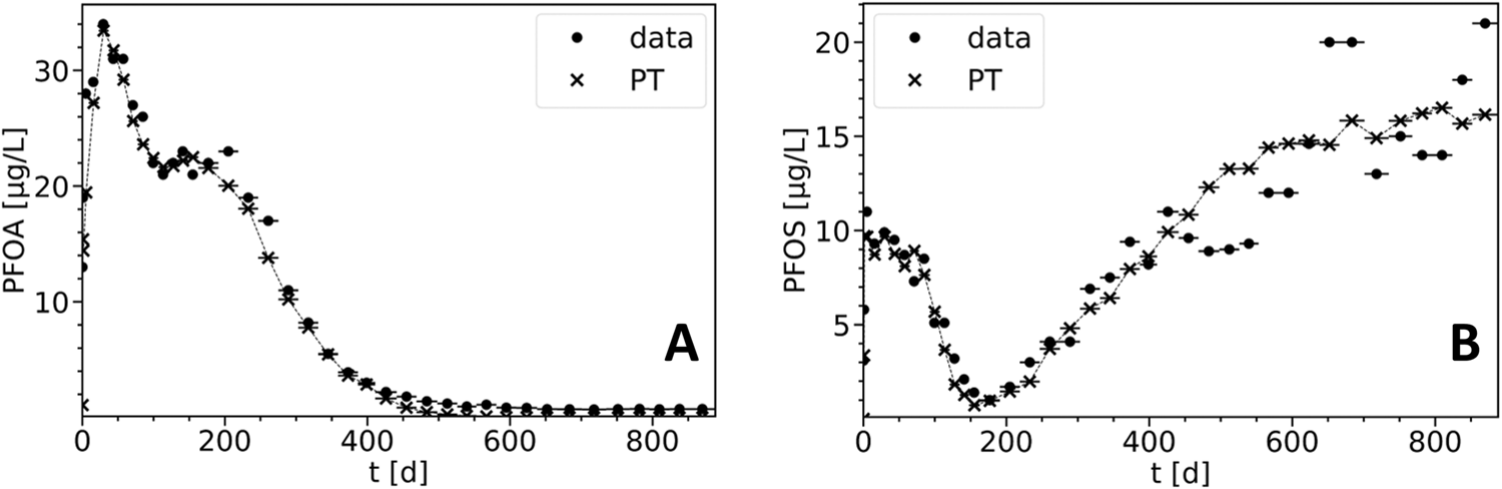
Observed data and PT-simulated PFOA (**A**) and PFOS (**B**) leachate concentrations in the lysimeter experiment using substance-specific transport parameters for the premature leaching (Eq. 15)

By employing substance-specific transport parameters for the premature leaching (Table 5, *R*_pre_), the fit of the PT results to the observed data was substantially improved for both PFOA and PFOS (Fig. 5). For PFOS, the prematurely leached mass fraction was 3.1%, and retardation coefficients of 4.7 (soil) and 2.7 (sand) were used. For PFOA, a much higher fraction of 15% was used, along with lower retardation coefficients (3.1/1.0). These findings imply that colloid-facilitated transport may not be the primary cause of the accelerated leaching observed in both leaching characteristics. This is evidenced by the unequal transport parameters for PFOS and PFOA, and the higher fraction of PFOA on colloids than that of PFOS, despite PFOA’s lower sorption affinity and lower initial soil content. However, colloid-facilitated transport may still play a significant role in the leaching of PFOS, but to a lesser extent for PFOA. This would align with PFOS’s higher sorption affinity and the anticipated greater importance of colloid-facilitated transport for this compound.

**Table 5:** Retardation coefficients in CM and PT simulations for the lysimeter experiment

	Model	*R*_pre_	*R*_s_	*R*_ia,s_	*R*_ia,sand_	*R*_eff_
PFOA	CM	–	3.8	2.4	3.8	5.7
PFOA	PT	3.1/1.0	4.0	5.5	5.5	8.0
PFOS	CM	–	17.0	51.2	82.4	75.6
PFOS	PT	4.7/2.7	17.0	63.0	63.0	71.5

*R*_pre_ is the retardation factor used for premature leaching, and *R*_eff_ denotes the effective retardation coefficient across the vertical lysimeter profile (excluding premature leaching)

Possibly, the initial saturated conditions and absence of AWI played a role in the premature leaching of PFAS. In this initial state, dissolved PFAS may have been transported with the pore water under quasi-saturated conditions without present AWI. After initial drainage, sorption to AWI led to a significant retardation. Additionally, when hydraulic conditions did not reach a quasi-constant flow and saturation state, flow rates were high, and rate-limited adsorption to AWI may have been relevant. This could account for the absence of premature leaching in the CM with equilibrium sorption to AWI. Most studies consider sorption to AWI to be instantaneous (Brusseau 2020; Silva et al. 2020). Stults et al. (2023) firstly reported rate-limited PFAS sorption to AWI due to higher *K*_ia_ values in experiments with lower flow rates. Guo et al. (2020) presented a model with a two-site approach for sorption to AWI including equilibrium and rate-limited partitioning. There, the equilibrium sorption was dominant. However, they did not investigate extreme flow events. Rate limitations of sorption to AWI may become important at high flow rates and fast saturation changes as it occurred in the initial phase of the lysimeter experiment.

In the PT, the adsorption and desorption rates (Table 4) used for simulating partitioning to the AWI were high enough to reach equilibrium in less than 3 h. If lower rates were employed (while maintaining the same ratio of rates), the fit to the steady-state leaching deteriorates, and the premature leaching converges with the steady-state leaching. Possibly, a more complex model incorporating rate-limited sorption to AWI and temporal and spatial resolution of AWI is needed to further investigate this, and it may be necessary to consider a concentration-dependent *K*_ia_.

It is important to note that the observed premature leaching is mainly a result of the experimental setup. Nevertheless, these observations suggest that extreme precipitation and soil structure disturbance can increase PFAS leaching. Generally, colloid-facilitated transport is more significant for PFAS with a high sorption affinity, i.e., long-chain PFAAs and many precursors. Additionally, rate-limited sorption to AWI may be relevant when saturation conditions change rapidly, i.e., at extreme rainfall or in sandy soils.

#### Comparison of CM and PT results

To compare the sorption parameters used in the CM and the PT, we calculated the retardation coefficients as defined by Eqs. (9) and (16) (Table 5). We found similar retardation coefficients in both model types, with sorption to AWI being the dominant retention process for PFOS. This demonstrates that the observed delayed leaching observed for PFOS was due to sorption to AWI in the sand layer. Moreover, this highlights the suitability of relatively straightforward Lagrangian models for examining PFAS leaching and assessing the relative importance of different retention mechanisms.

In the CM, the retardation is higher in the sand layer compared to the soil layer (Table 5), mainly attributed to lower saturation levels, which is consistent with the findings of Guo et al. (2020). In the PT, the retardation due to sorption to AWI (*R*_ia,s_) is 3.7 times higher than the retardation due to sorption to the soil (*R*_s_). *R*_ia,s_ is lower in the CM (51.2 vs. 63), while *R*_ia,sand_ is higher (82.4 vs. 63) which is a result of the saturation-dependent AWI in the CM. Overall, this leads to similar effective retardation coefficients, *R*_eff_, of 75.6 in the CM and 71.5 in the PT, demonstrating their general agreement.

The retardation coefficients obtained in this study are within the range of values reported in other simulation studies. In a study by Silva et al. (2020), 96% of PFOS retardation was attributed to AWI. Brusseau (2018) employed concentration-dependent *K*_ia_ and showed that the retardation coefficient of PFOS exceed 200 for concentrations less than 10 µg/L. To the best of the authors’ knowledge, this study presents the highest experimentally observed retardation coefficients due to AWI. Reported retardation coefficients in previous experimental studies were below 10 (Lyu et al. 2018; Brusseau et al. 2019b, 2021; Abraham et al. 2022; Stults et al. 2022). This can be attributed to the relatively low saturation conditions in the current study, which enhances the importance of sorption to AWI. For instance, Brusseau et al. (2021) conducted experiments at a saturation level of 0.68, while the saturation in the sand layer of the present study is approximately 0.19.

At the end of the lysimeter experiment, the measured residual concentrations of PFOA were low (3.3–3.9 µg/kg), while PFOS concentrations remained high and displayed a distinct concentration profile in the soil layer, with lower concentrations at the top (59 µg/kg) and higher concentrations at the bottom layer (230 µg/kg) (Table S2). The observed residual concentrations in the sand layer of 21 µg/kg PFOS were attributed to sorption on AWI.

**Fig. 6:**
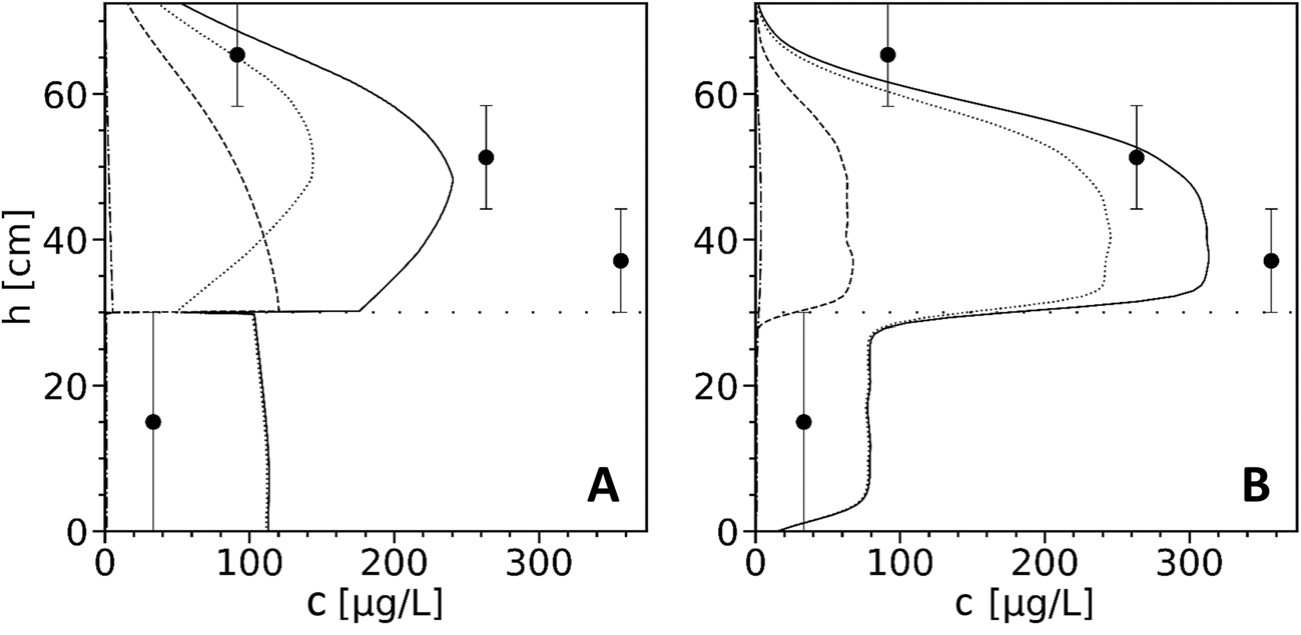
PFOS concentration profile (mass per bulk volume) in CM (**A**) and PT (**B**) simulations at the end of the lysimeter experiment (*t* = 888 d), showing partitioning to the liquid phase (dash-dotted line), the solid phase (dashed line), and the AWI (dotted line). The solid lines represent the total concentration. Solid circles with error bars denote the experimental data. The horizontal dotted line at *h* = 30 cm delineates the transition between soil and sand layers

Figure 6 presents the measured and predicted residual PFOS concentrations in the soil matrix, including partitioning between phases, as depicted by the PT and the CM. While both models emphasize the important role of PFAS sorption to the AWI, the models diverge in their quantification of this process. In the PT model, the majority of PFOS is predicted to be sorbed to the AWI, in contrast to the CM where sorption to the solid phase is approximately equal to sorption to the AWI. This discrepancy between the models was expected to some extent due to the lower retardation factor (*R*_ia,s_) used in the CM. However, the magnitude of the difference observed was not anticipated suggesting that the more detailed calculation of the AWI in the CM introduces additional variability into the partitioning of PFOS between phases. This is further underscored by the PT model results showing that the ratio of PFOS sorbed to the AWI versus the solid phase is reflected in the ratio of their corresponding retardation factors. Contrarily, this direct correlation is not observed in the CM, likely due to the model’s more detailed representation of the AWI.

Furthermore, notable differences were observed in the two models regarding the concentration profile on the AWI. In the PT model, AWI sorption reflected the profile of solid sorption, presenting a coherent pattern between these two sorption mechanisms. Conversely, in the CM, the peak of the AWI sorption was found in the middle of the soil layer. This central peak can be attributed to the model’s configuration, where an increasing AWI area towards the top of the soil profile shifts the peak of the absolute mass sorbed to the AWI. The resulting total concentrations both in magnitude and shape across the profile were more adequately predicted by the PT compared to the CM. These observations suggest that the AWI area may not exhibit significant variability along the vertical soil profile, contrary to the depiction in the CM. Instead, it is possible that the AWI area remains relatively constant across the vertical profile, aligning the profiles of AWI sorption and solid phase sorption as suggested by the PT simulations.

Both models overestimated the PFOS concentration in the sand layer, but the PT was closer to the measured value. Notably, the PT captured more accurately the pronounced difference in concentrations between the soil and sand layers, aligning more consistently with the experimental observations. Possibly, AWI sorption in the sand layer is slightly overestimated by both models indicating that sorption to sand grains also plays a significant role as a retention mechanism. Nevertheless, the predicted total concentration would remain relatively stable, as the modeled retardation of PFOS transport in the sand layer is consistent with the experimental observations.

These results are noteworthy because the simpler model, in terms of AWI resolution, was significantly closer to the observed data. Our analysis suggests that the simplified representation of AWI sorption and hydraulic properties, as implemented in the PT, enhanced the fit to the experimental observations. The PT model employs average saturation values for the sand and soil layers, respectively, while the CM updates saturation continuously resulting in a common variability across the vertical profile. This difference has consequential effects on the modeling of sorption to AWI between the two models. In the CM, the AWI area increases with the vertical height, while the magnitude of AWI sorption is constant within the soil and sand layers in the PT.

The discrepancies could be partly due to an inadequate capillary-pressure–saturation relationship or an unsuitable functional relation between saturation and AWI area for the used solid materials in the CM. While our study did not explore various relationships between saturation and AWI, it is generally understood that the AWI area would increase with decreasing saturation. Consequently, independent of the precise specifications of the capillary-pressure–saturation relationship, the resulting peak of AWI sorption in the CM would likely be positioned in the center of the soil layer. This is expected due to the general trend of decreasing saturation and, consequently, increasing AWI area towards the top of the soil profile in the CM’s configuration. However, the experimental observations and the PT results challenge this relationship, as the highest PFAS concentrations were observed at the bottom of the soil layer. These intricate findings highlight the necessity for further research on PFAS partitioning between phases under varying saturation conditions. Overall, it is unlikely that the models overestimated AWI sorption, as the observed delayed leaching was well-reproduced, and the sorption parameters are consistent with reported values as discussed above.

## Conclusions

This study illuminates key sorption processes influencing PFAS transport, highlighting the relevance of equilibrium and rate-limited sorption to soil and AWI, and examining the role of colloid-facilitated transport. Equilibrium sorption models were found sufficient to estimate Freundlich parameters, while rate-limited sorption was necessary for capturing tailing and prolonged leaching. Notably, sorption parameters varied between experimental setups, particularly for PFOA, hinting at biotransformation influences in lysimeter results.

Our PT simulations demonstrated the potential for colloid-facilitated transport to cause premature breakthroughs, highlighting it as an important process under extreme flow conditions. However, observed substance-specific best-fit parameters for PFOA and PFOS suggest additional kinetic processes at play. We proposed the hypothesis that rate-limited sorption to AWI during non-steady states may be relevant, delaying steady-state attainment and possibly inducing premature breakthrough—an outcome absent in the CM predictions with equilibrium AWI sorption. This hypothesis requires further focused research.

Moreover, we highlighted random walk particle tracking as an advantageous, alternative modeling approach for PFAS leaching. Its practical framework, allowing mobile/immobile state definition and phase transition rates, facilitated distinct analysis of transport/retention mechanisms and enabled the simulation of observed premature leaching as a separate process. Notably, the PT aligned more closely with experimental data than the CM despite AWI sorption simplifications challenging the configurations of AWI sorption in the CM.

Both the CM and PT demonstrated that AWI sorption can be the dominant retention mechanism for long-chain PFAAs under variably saturated conditions, affirming the value of variably saturated studies that incorporate AWI sorption for precise PFAS leaching predictions and contaminated site management. Both models showed that the drastic increase in PFOS concentrations at relatively late time was not due to transformation of precursors, but predominantly due to interplay of sorption processes.

### Supplementary Information

Below is the link to the electronic supplementary material.

Supplementary file1 (PDF 751 KB)

## Data Availability

The data will be made available on request.

## References

[CR1] Abraham JEF, Mumford KG, Patch DJ, Weber KP (2022). Retention of PFOS and PFOA mixtures by trapped gas bubbles in porous media. Environ Sci Technol.

[CR2] Adamson AW, Gast AP (1967) Physical chemistry of surfaces. Interscience, No. 150. 10.1126/science.160.3824.179.a

[CR3] Ahlstrom SW, Foote HP, Arnett RC, Cole CR, Serne RJ (1977) Multicomponent mass transport model: theory and numerical implementation (discrete-parcel-random-walk version). In: Battelle Pacific Northwest Labs., Richland, Wash. (USA). 10.2172/7083383.

[CR4] Bierbaum T, Klaas N, Braun J, Nürenberg G, Lange FT, Haslauer C (2023). Immobilization of per- and polyfluoroalkyl substances (PFAS): comparison of leaching behavior by three different leaching tests. Sci Total Environ.

[CR5] Borthakur A, Cranmer BK, Dooley GP, Blotevogel J, Mahendra S, Mohanty SK (2021). Release of soil colloids during flow interruption increases the pore-water PFAS concentration in saturated soil. Environ Pollut (Barking, Essex : 1987).

[CR6] Boso F, Bellin A, Dumbser M (2013). Numerical simulations of solute transport in highly heterogeneous formations: a comparison of alternative numerical schemes. Adv Water Resour.

[CR7] Bradford SA, Wang Y, Torkzaban S, Šimůnek J (2015). Modeling the release of E. coli D21g with transients in water content. Water Resour Res.

[CR8] Bräunig J, Baduel C, Barnes CM, Mueller JF (2019). Leaching and bioavailability of selected perfluoroalkyl acids (PFAAs) from soil contaminated by firefighting activities. The Sci Total Environ.

[CR9] Brusseau ML (2018). Assessing the potential contributions of additional retention processes to PFAS retardation in the subsurface. Sci Total Environ.

[CR10] Brusseau ML (2020). Simulating PFAS transport influenced by rate-limited multi-process retention. Water Res.

[CR11] Brusseau ML, van Glubt S (2019). The influence of surfactant and solution composition on PFAS adsorption at fluid-fluid interfaces. Water Res.

[CR12] Brusseau ML, Jessup RE, Rao PSC (1991). Nonequilibrium sorption of organic chemicals: elucidation of rate-limiting processes. Environ Sci Technol.

[CR13] Brusseau ML, Khan N, Wang Y, Yan N, van Glubt S, Carroll KC (2019). Nonideal Transport and extended elution tailing of PFOS in soil. Environ Sci Technol.

[CR14] Brusseau ML, Yan N, van Glubt S, Wang Y, Chen W, Lyu Y, Dungan B, Carroll KC, Holguin FO (2019). Comprehensive retention model for PFAS transport in subsurface systems. Water Res.

[CR15] Brusseau ML, Guo B, Huang D, Yan N, Lyu Y (2021). Ideal versus nonideal transport of PFAS in unsaturated porous media. Water Res.

[CR16] Buck RC, Franklin J, Berger U, Conder JM, Cousins IT, de Voogt P, Jensen AA, Kannan K, Mabury SA, van Leeuwen SPJ (2011). Perfluoroalkyl and polyfluoroalkyl substances in the environment: terminology, classification, and origins. Integr Environ Assess Manag.

[CR17] Cai W, Navarro DA, Du J, Ying G, Yang B, McLaughlin MJ, Kookana RS (2022). Increasing ionic strength and valency of cations enhance sorption through hydrophobic interactions of PFAS with soil surfaces. Sci Total Environ.

[CR18] Campos Pereira H, Ullberg M, Kleja DB, Gustafsson JP, Ahrens L (2018). Sorption of perfluoroalkyl substances (PFASs) to an organic soil horizon - effect of cation composition and pH. Chemosphere.

[CR19] Dentz M, Berkowitz B (2003) Transport behavior of a passive solute in continuous time random walks and multirate mass transfer. Water Resour Res 39(5). 10.1029/2001WR001163

[CR20] Du Z, Deng S, Bei Y, Huang Q, Wang B, Huang J, Yu G (2014). Adsorption behavior and mechanism of perfluorinated compounds on various adsorbents–a review. J Hazard Mater.

[CR21] Fabregat-Palau J, Vidal M, Rigol A (2021). Modelling the sorption behaviour of perfluoroalkyl carboxylates and perfluoroalkane sulfonates in soils. Sci Total Environ.

[CR22] Freundlich H (1907) Über die Adsorption in Lösungen. Zeitschrift für Physikalische Chemie 57U(1). 10.1515/zpch-1907-5723

[CR23] Gagliano E, Sgroi M, Falciglia PP, Vagliasindi FGA, Roccaro P (2020). Removal of poly- and perfluoroalkyl substances (PFAS) from water by adsorption: role of PFAS chain length, effect of organic matter and challenges in adsorbent regeneration. Water Res.

[CR24] Guelfo JL, Higgins CP (2013). Subsurface transport potential of perfluoroalkyl acids at aqueous film-forming foam (AFFF)-impacted sites. Environ Sci Technol.

[CR25] Guelfo JL, Wunsch A, McCray J, Stults JF, Higgins CP (2020). Subsurface transport potential of perfluoroalkyl acids (PFAAs): column experiments and modeling. J Contam Hydrol.

[CR26] Guo B, Zeng J, Brusseau ML (2020) A mathematical model for the release, transport, and retention of per- and polyfluoroalkyl substances (PFAS) in the vadose zone. Water Resour Res 56(2). 10.1029/2019WR026667 10.1029/2019wr026667 PMC7673302 33223573

[CR27] Haggerty R, Gorelick SM (1995). Multiple-rate mass transfer for modeling diffusion and surface reactions in media with pore-scale heterogeneity. Water Resour Res.

[CR28] Hansen SK, Berkowitz B (2020). Aurora: A non-Fickian (and Fickian) particle tracking package for modeling groundwater contaminant transport with MODFLOW. Environ Model Softw.

[CR29] Hansen SK, Berkowitz B (2020b) Modeling non-Fickian solute transport due to mass transfer and physical heterogeneity on arbitrary groundwater velocity fields. Water Resour Res 56(10). 10.1029/2019WR026868

[CR30] Hansen SK, Vesselinov VV (2018). Local equilibrium and retardation revisited. Groundwater.

[CR31] Hedia AM, Abd-Elmegeed MA, Hassan AE (2021) Using particle tracking to simulate contaminant transport in the presence of colloids and bacteria. Arab J Geosci 14(19). 10.1007/s12517-021-08306-6

[CR32] Hellsing MS, Josefsson S, Hughes AV, Ahrens L (2016). Sorption of perfluoroalkyl substances to two types of minerals. Chemosphere.

[CR33] Henri CV, Diamantopoulos E (2022) Unsaturated transport modeling: random-walk particle-tracking as a numerical-dispersion free and efficient alternative to Eulerian methods. J Adv Model Earth Syst 14(9). 10.1029/2021MS002812

[CR34] Henri CV, Fernàndez-Garcia D (2015). A random walk solution for modeling solute transport with network reactions and multi-rate mass transfer in heterogeneous systems: impact of biofilms. Adv Water Resour.

[CR35] Higgins CP, Luthy RG (2006). Sorption of perfluorinated surfactants on sediments. Environ Sci Technol.

[CR36] Just H, Göckener B, Lämmer R, Wiedemann-Krantz L, Stahl T, Breuer J, Gassmann M, Weidemann E, Bücking M, Kowalczyk J (2022). Degradation and plant transfer rates of seven fluorotelomer precursors to perfluoroalkyl acids and F-53B in a soil-plant system with maize (Zea mays L.). J Agricult Food Chem.

[CR37] Kotthoff M, Fliedner A, Rüdel H, Göckener B, Bücking M, Biegel-Engler A, Koschorreck J (2020). Per- and polyfluoroalkyl substances in the German environment - levels and patterns in different matrices. Sci Total Environ.

[CR38] Lee H, Tevlin AG, Mabury SA, Mabury SA (2014). Fate of polyfluoroalkyl phosphate diesters and their metabolites in biosolids-applied soil: biodegradation and plant uptake in greenhouse and field experiments. Environ Sci Technol.

[CR39] Li Y, Oliver DP, Kookana RS (2018). A critical analysis of published data to discern the role of soil and sediment properties in determining sorption of per and polyfluoroalkyl substances (PFASs). Sci Total Environ.

[CR40] Liu C, Liu J (2016). Aerobic biotransformation of polyfluoroalkyl phosphate esters (PAPs) in soil. Environ Pollut (Barking, Essex : 1987).

[CR41] Loschko M, Wöhling T, Rudolph DL, Cirpka OA (2016). Cumulative relative reactivity: a concept for modeling aquifer-scale reactive transport. Water Resour Res.

[CR42] Lv X, Sun Y, Ji R, Gao B, Wu J, Lu Q, Jiang H (2018). Physicochemical factors controlling the retention and transport of perfluorooctanoic acid (PFOA) in saturated sand and limestone porous media. Water Res.

[CR43] Lyu Y, Brusseau ML, Chen W, Yan N, Fu X, Lin X (2018). Adsorption of PFOA at the air-water interface during transport in unsaturated porous media. Environ Sci Technol.

[CR44] Maizel AC, Shea S, Nickerson A, Schaefer C, Higgins CP (2021). Release of per- and polyfluoroalkyl substances from aqueous film-forming foam impacted soils. Environ Sci Technol.

[CR45] Martin JW, Asher BJ, Beesoon S, Benskin JP, Ross MS (2010). PFOS or PreFOS? Are perfluorooctane sulfonate precursors (PreFOS) important determinants of human and environmental perfluorooctance sulfonate (PFOS) exposure?. JEnviron Monitor.

[CR46] Milinovic J, Lacorte S, Vidal M, Rigol A (2015). Sorption behaviour of perfluoroalkyl substances in soils. Sci Total Environ.

[CR47] Nguyen TMH, Bräunig J, Thompson K, Thompson J, Kabiri S, Navarro DA, Kookana RS, Grimison C, Barnes CM, Higgins CP, McLaughlin MJ, Mueller JF (2020). Influences of chemical properties, soil properties, and solution ph on soil-water partitioning coefficients of per- and polyfluoroalkyl substances (PFASs). Environ Sci Technol.

[CR48] Nguyen TMH, Bräunig J, Kookana RS, Kaserzon SL, Knight ER, Vo HNP, Kabiri S, Navarro DA, Grimison C, Riddell N, Higgins CP, McLaughlin MJ, Mueller JF (2022). Assessment of mobilization potential of per- and polyfluoroalkyl substances for soil remediation. Environ Sci Technol.

[CR49] Toward a new comprehensive global database of per- and polyfluoroalkyl substances (PFASs): summary report on updating the OECD 2007 list of per- and polyfluoroalkyl substances (PFASs). ENV/JM/MONO

[CR50] Richards LA (1931). Capillary conduction of liquids through porous mediums. Physics.

[CR51] Röhler K, Haluska AA, Susset B, Liu B, Grathwohl P (2021). Long-term behavior of PFAS in contaminated agricultural soils in Germany. J Contam Hydrol.

[CR52] Schaefer CE, Culina V, Nguyen D, Field J (2019). Uptake of poly- and perfluoroalkyl substances at the air-water interface. Environ Sci Technol.

[CR53] Schaefer CE, Hooper J, Modiri-Gharehveran M, Drennan DM, Beecher N, Lee L (2022). Release of poly- and perfluoroalkyl substances from finished biosolids in soil mesocosms. Water Res.

[CR54] Schaefer CE, Nguyen D, Christie E, Shea S, Higgins CP, Field J (2022b) Desorption isotherms for poly- and perfluoroalkyl substances in soil collected from an aqueous film-forming foam source area. J Environ Eng 148(1). 10.1061/(ASCE)EE.1943-7870.0001952

[CR55] Shao M, Ding G, Zhang J, Wei L, Xue H, Zhang N, Li Y, Chen G, Sun Y (2016). Occurrence and distribution of perfluoroalkyl substances (PFASs) in surface water and bottom water of the Shuangtaizi Estuary, China. Environ Pollut (Barking, Essex : 1987).

[CR56] Silva JAK, Martin WA, Johnson JL, McCray JE (2019). Evaluating air-water and NAPL-water interfacial adsorption and retention of perfluorocarboxylic acids within the Vadose zone. J Contam Hydrol.

[CR57] Silva JAK, Šimůnek J, McCray JE (2020). A modified HYDRUS model for simulating PFAS transport in the vadose zone. Water.

[CR58] Silva JAK, Martin WA, McCray JE (2021). Air-water interfacial adsorption coefficients for PFAS when present as a multi-component mixture. J Contam Hydrol.

[CR59] Silva JA, Guelfo JL, Šimůnek J, McCray JE (2022). Simulated leaching of PFAS from land-applied municipal biosolids at agricultural sites. J Contam Hydrol.

[CR60] Silva JAK, Šimůnek J, McCray JE (2022). Comparison of methods to estimate air-water interfacial areas for evaluating PFAS transport in the vadose zone. J Contam Hydrol.

[CR61] Sima MW, Jaffé PR (2021). A critical review of modeling poly- and perfluoroalkyl substances (PFAS) in the soil-water environment. Sci Total Environ.

[CR62] Šimůnek J, Genuchten MT, Šejna M (2008). Development and applications of the HYDRUS and STANMOD software packages and related codes. Vadose Zone J.

[CR63] Stahl T, Mattern D, Brunn H (2011) Toxicology of perfluorinated compounds. Environ Sci Eur 23(1). 10.1186/2190-4715-23-38

[CR64] Steenland K, Fletcher T, Savitz DA (2010). Epidemiologic evidence on the health effects of perfluorooctanoic acid (PFOA). Environ Health Perspect.

[CR65] Stults JF, Choi YJ, Schaefer CE, Illangasekare TH, Higgins CP (2022). Estimation of transport parameters of perfluoroalkyl acids (PFAAs) in unsaturated porous media: critical experimental and modeling improvements. Environ Sci Technol.

[CR66] Stults JF, Choi YJ, Rockwell C, Schaefer CE, Nguyen DD, Knappe DRU, Illangasekare TH, Higgins CP (2023). Predicting concentration- and ionic-strength-dependent air-water interfacial partitioning parameters of PFASs using quantitative structure-property relationships (QSPRs). Environ Sci Technol.

[CR67] van Genuchten MT (1980). A closed-form equation for predicting the hydraulic conductivity of unsaturated soils. Soil Sci Soc Am J.

[CR68] van Genuchten MT, Wagenet RJ (1989). Two-site/two-region models for pesticide transport and degradation: theoretical development and analytical solutions. Soil Sci Soc Am J.

[CR69] van Glubt S, Brusseau ML, Yan N, Huang D, Khan N, Carroll KC (2021). Column versus batch methods for measuring PFOS and PFOA sorption to geomedia. Environ Pollut (Barking, Essex : 1987).

[CR70] Vecitis CD, Park H, Cheng J, Mader BT, Hoffmann MR (2008). Enhancement of perfluorooctanoate and perfluorooctanesulfonate activity at acoustic cavitation bubble interfaces. J Phys Chem C.

[CR71] Weidemann E, Lämmer R, Stahl T, Göckener B, Bücking M, Breuer J, Kowalczyk J, Just H, Boeddinghaus RS, Gassmann M (2022). Leaching and transformation of perfluoroalkyl acids and polyfluoroalkyl phosphate diesters in unsaturated soil column studies. Environ Toxicol Chem.

[CR72] Xiao X, Ulrich BA, Chen B, Higgins CP (2017). Sorption of poly- and perfluoroalkyl substances (PFASs) relevant to aqueous film-forming foam (AFFF)-impacted groundwater by biochars and activated carbon. Environ Sci Technol.

[CR73] Xiao F, Jin B, Golovko SA, Golovko MY, Xing B (2019). Sorption and desorption mechanisms of cationic and zwitterionic per- and polyfluoroalkyl substances in natural soils: thermodynamics and hysteresis. Environ Sci Technol.

[CR74] Yi S, Harding-Marjanovic KC, Houtz EF, Gao Y, Lawrence JE, Nichiporuk RV, Iavarone AT, Zhuang W-Q, Hansen M, Field JA, Sedlak DL, Alvarez-Cohen L (2018). Biotransformation of AFFF component 6:2 fluorotelomer thioether amido sulfonate generates 6:2 fluorotelomer thioether carboxylate under sulfate-reducing conditions. Environ Sci Technol Lett.

[CR75] Yin T, Te SH, Reinhard M, Yang Y, Chen H, He Y, Gin KY-H (2018). Biotransformation of Sulfluramid (N-ethyl perfluorooctane sulfonamide) and dynamics of associated rhizospheric microbial community in microcosms of wetland plants. Chemosphere.

[CR76] Zeng C, Atkinson A, Sharma N, Ashani H, Hjelmstad A, Venkatesh K, Westerhoff P (2020) Removing per- and polyfluoroalkyl substances from groundwaters using activated carbon and ion exchange resin packed columns. AWWA Water Sci 2(1). 10.1002/aws2.1172

[CR77] Zhang S, Szostek B, McCausland PK, Wolstenholme BW, Lu X, Wang N, Buck RC (2013). 6:2 and 8:2 fluorotelomer alcohol anaerobic biotransformation in digester sludge from a WWTP under methanogenic conditions. Environ Sci Technol.

[CR78] Zhang S, Lu X, Wang N, Buck RC (2016). Biotransformation potential of 6:2 fluorotelomer sulfonate (6:2 FTSA) in aerobic and anaerobic sediment. Chemosphere.

