# Supplementary material for: Investigating rate-limited sorption, sorption to air–water interfaces, and colloid-facilitated transport during PFAS leaching
Source: Environ Sci Pollut Res Int. 2023 Nov 13;30(58):121529–47. doi: 10.1007/s11356-023-30811-2 (PMC10724089; doi:10.1007/s11356-023-30811-2)
Supplement: Supplementary file 1 — Supplementary file1 (PDF 751 KB) [file 11356_2023_30811_MOESM1_ESM.pdf]

Investigating Rate-Limited Sorption, Sorption to Air-Water Interfaces, and Colloid-Facilitated Transport During PFAS Leaching

Thomas Bierbaum*[1], Scott K. Hansen[2], Bikash Poudel[1], Claus Haslauer[1]

[1]: *University of Stuttgart, Institute for Modelling Environmental Systems (IWS), Research Facility for Subsurface Remediation (VEGAS), Pfaffenwaldring 61, 70569 Stuttgart, Germany*

[2]: *Ben-Gurion University of the Negev, Zuckerberg Institute for Water Research, Midreshet Ben-Gurion 8499000, Israel*

**Corresponding author:*

E-mail address: thomas.bierbaum@iws.uni-stuttgart.de
University of Stuttgart, Institute for Modelling Environmental Systems (IWS), Research Facility for Subsurface Remediation (VEGAS), Pfaffenwaldring 61, 70569 Stuttgart, Germany

Tables

Table S1 PFAS concentrations observed in experiments. Mean soil concentrations were 45 µg/kg PFOA and 186 µg/kg PFOS. m_{tot} is the sum of the total mass observed in the leachates and the residual soil concentrations at the end of the experiments, normalized to 1 kg soil. m_{out} is the cumulated leached mass. Consequently, the residual soil concentrations are the differences between m_{tot} and m_{out}.

	m_{tot} [µg/kg]	m_{out} [µg/kg]	m_{tot} [µg/kg]	m_{out} [µg/kg]
	PFOA	PFOA	PFOS	PFOS
Col1	48.6	44.7	174.8	169.5
Col2	39.6	37.8	156.8	154.9
Col3	28.1	25.1	108.2	104.2
Col4	42.3	39.1	153.8	150.2
Lysimeter	46.6	43.0	205	36.5

Table S2 PFAS soil concentrations (µg/kg) at the end of the lysimeter experiment.

	PFOA	PFOS
Top soil layer	3.7	59
Center soil layer	3.9	170
Bottom soil layer	3.3	230
Sand layer	<1	21

Table S3 Experimental parameters in column and lysimeter experiments (m_s: soil mass, ρ_b: bulk density, n: porosity, s_w: saturation, pv: pore volume, t_c: contact time, q: seepage velocity, t_{end}: total operating time, LS_{end}: total reached liquid-to-solid ratio). s_w value of the lysimeter experiment is the mean saturation suggested by the continuum model (CM). See also Bierbaum et al. (2023).

Experiment	m_s [kg]	ρ_b [kg/L]	n [-]	s_w [-]	pv [L]	t_c [h]	q [cm/d]	t_{end} [d]	LS_{end} [L/kg]
Col1	3.9	1.55	0.42	1	1.06	5	58.4	20	19
Col2	4.0	1.58	0.4	1	1.03	11	31.7	142	71
Col3	4.0	1.55	0.41	1	1.05	18	19.5	160	50
Col4	3.7	1.44	0.45	1	1.16	48	9.0	159	25
Lysimeter	186.4	1.55	0.41	0.57	50	≈ 860	0.311	888	4.1

Table S4 van Genuchten parameters of N-1 used in Hydrus.

	θ_r	θ_s	α [1/cm]	n	K [cm/d]
N-1	0.1	0.41	0.0265448	3.5314	156
Sand	0.045	0.4	0.145	2.68	5700

Table S5 Initial concentrations (*c*: liquid concentration, s_k: sorbed concentration of the kinetic phase) in numerical simulations with equilibrium sorption and two-site sorption.

PFAS	Experiment	Sorption model	c_0 [µg/cm³]	$S_{k,0}$ [µg/g]	*RMSLE*
PFOA	Col1	*equ*	0.0481	-	0.258
PFOA	Col2	*equ*	0.04	-	0.143
PFOA	Col3	*equ*	0.028	-	0.434
PFOA	Col4	*equ*	0.0403	-	0.189
PFOA	Col1	*2ss*	0.032	0.0101	0.325
PFOA	Col2	*2ss*	0.0243	0.0109	0.066
PFOA	Col3	*2ss*	0.019	0.0054	0.414
PFOA	Col4	*2ss*	0.030	0.0051	0.062
PFOS	Col1	*equ*	0.0359	-	0.219
PFOS	Col2	*equ*	0.032	-	0.188
PFOS	Col3	*equ*	0.021	-	0.407
PFOS	Col4	*equ*	0.0309	-	0.145
PFOS	Col1	*2ss*	0.035	0.00799	0.184
PFOS	Col2	*2ss*	0.032	0.0053	0.339
PFOS	Col3	*2ss*	0.021	0.0077	0.424
PFOS	Col4	*2ss*	0.03	0.0098	0.144
PFOA	Lys (col params)	*2ss*	0.035	0.00470	0.254
PFOA	Lys	*2ss*	0.035	0.00470	0.281
PFOA	Lys	*equ + AWI*	0.0368	-	0.538
PFOA	Lys	*2ss + AWI*	0.035	0.00470	0.434
PFOS	Lys	*equ*	0.0368	-	1.158

Table S6 Sorbed concentrations (µg/kg) of the kinetic phase (s_k) at time t_{end} (Table S3) corresponding to the end of the respective experiment.

	Col1	Col2	Col3	Col4	Lysimeter
PFOA	8.3	2.6	1.1	1.0	2.1
PFOS	7.5	2.7	3.7	4.8	-

Figures

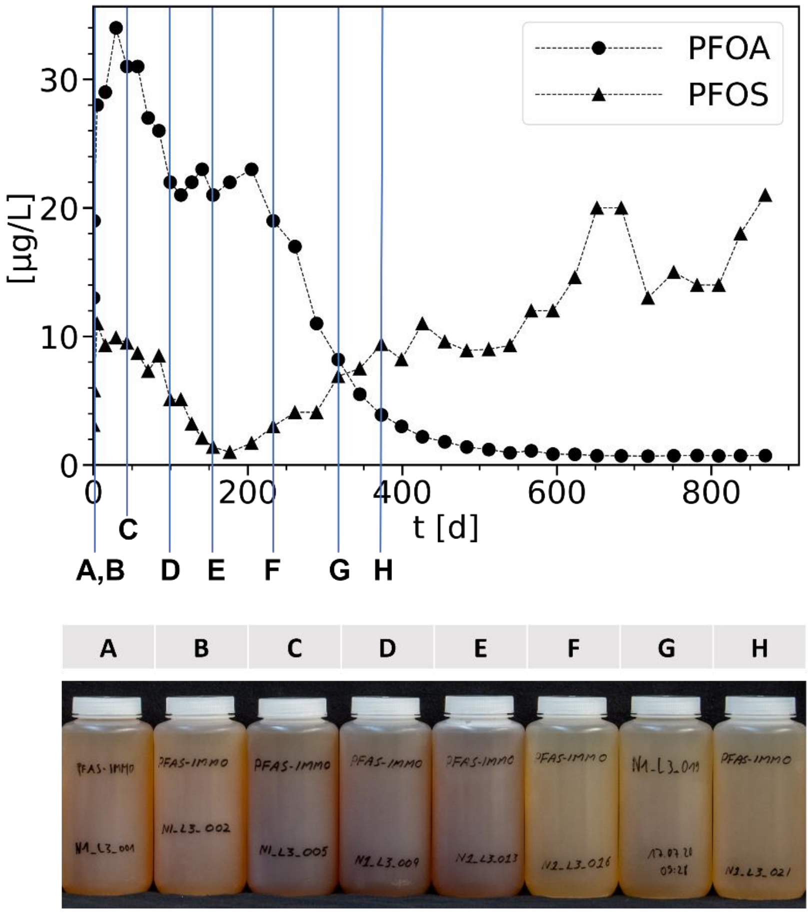

Fig. S1 Selected leachate samples (A - H) indicating leaching of colloids and corresponding PFOA and PFOS leachate concentrations in the lysimeter experiment.

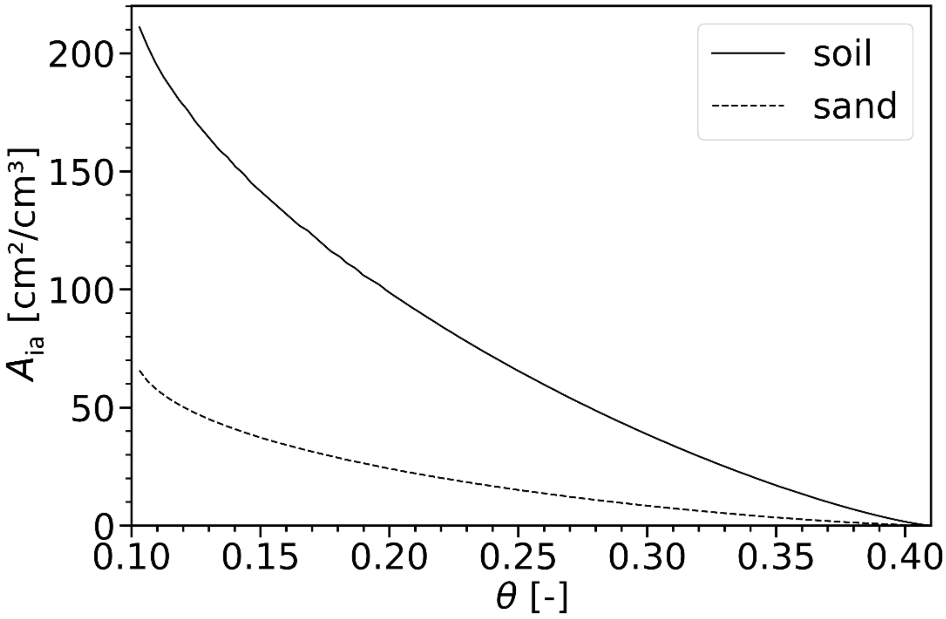

Fig. S2 Relation of air-water interfacial area (A_{ia}) and water content (θ) used in the continuum model (CM).

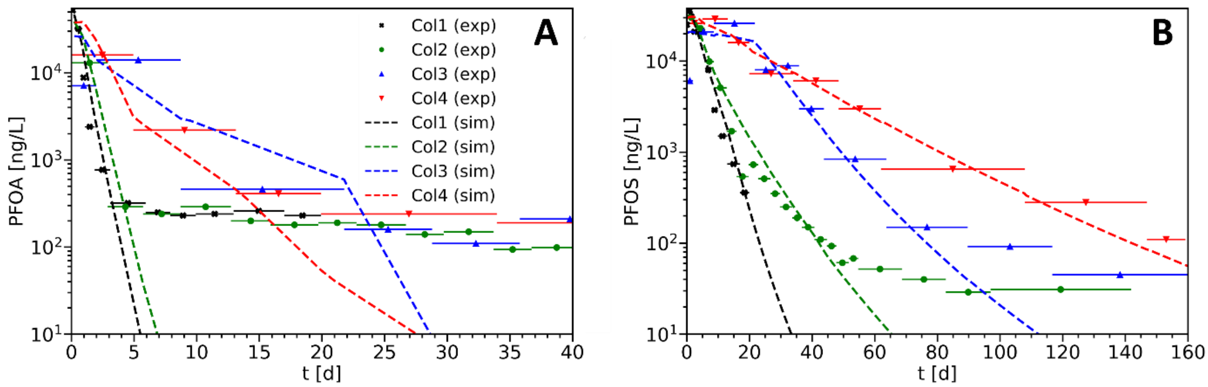

Fig. S3 Simulated (dashed lines) leachate concentrations of PFOA (A) and PFOS (B) in the saturated column experiments with varying flow rates using equilibrium sorption (Freundlich). Horizontal bars in the observed data (markers) represent their corresponding sampling intervals. Different colors and markers denote individual experiments.

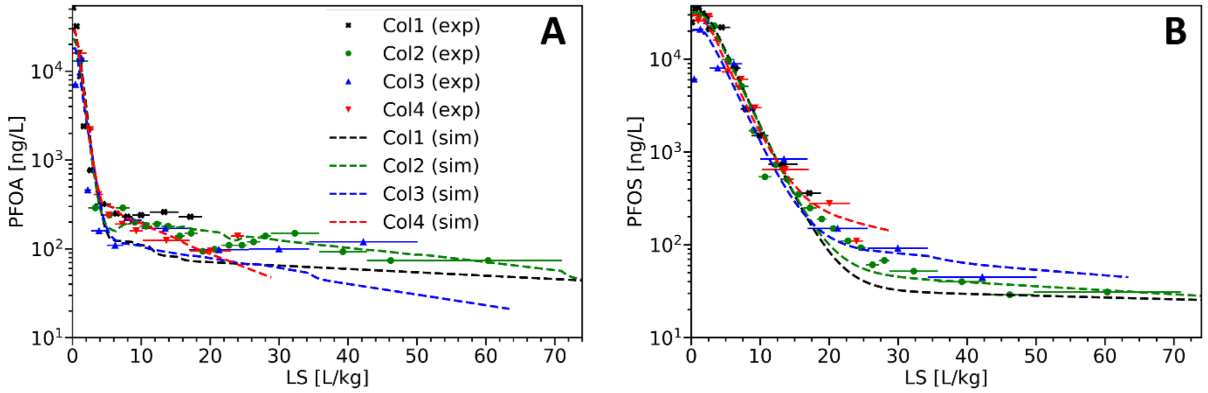

Fig. S4 Observed (markers with horizontal bars) and *2ss* model-simulated (dashed lines) leachate concentrations of PFOA (A) and PFOS (B) in the saturated column experiments with varying flow rates over the liquid-to-solid ratio (*LS*). Horizontal bars in the observed data represent their corresponding sampling intervals. Different colors and markers denote individual experiments.

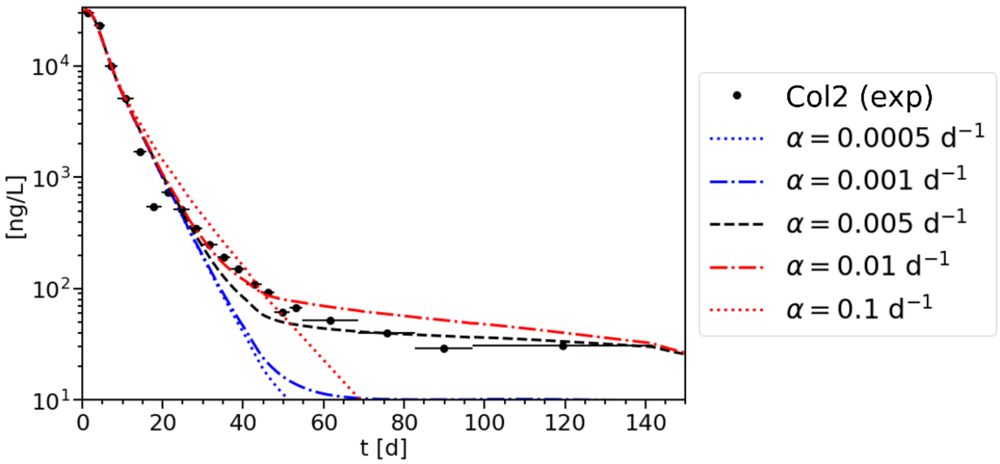

Fig. S5 Sensitivity analysis of α in the 2ss model simulating PFOS leachate concentrations in the column experiment Col2.

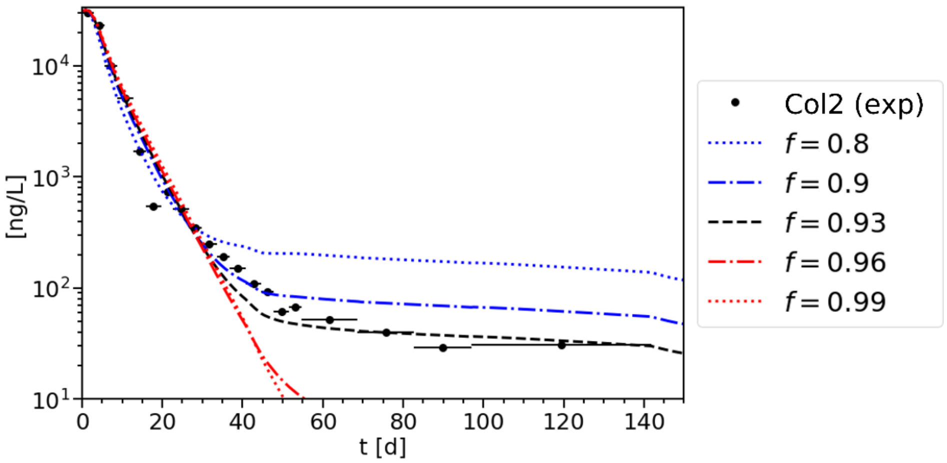

Fig. S6 Sensitivity analysis of f in the 2ss model simulating PFOS leachate concentrations in the column experiment Col2.

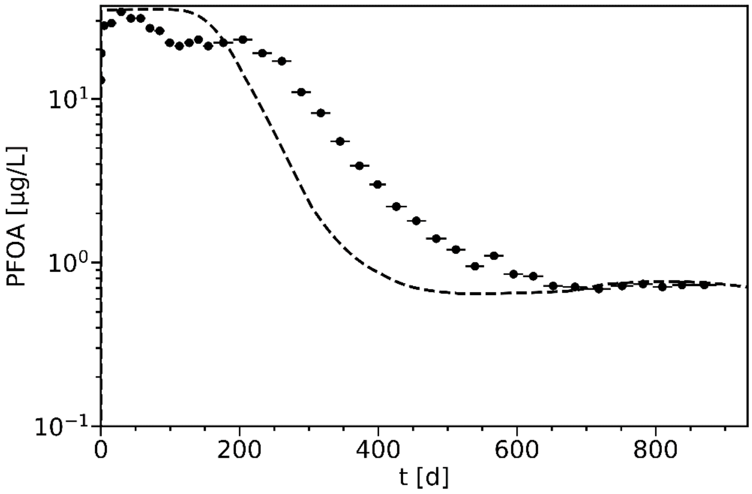

Fig. S7 Simulated (dashed line) and observed (●) PFOA concentrations in the lysimeter experiment. Long tailing was simulated with a source term representing transformation of precursors and production of PFOA. Sorption to AWI was not included in the model, consequently, retardation is lower and breakthrough is generally faster compared to the observed leaching.
